# Retromer Is Essential for Autophagy-Dependent Plant Infection by the Rice Blast Fungus

**DOI:** 10.1371/journal.pgen.1005704

**Published:** 2015-12-10

**Authors:** Wenhui Zheng, Jie Zhou, Yunlong He, Qiurong Xie, Ahai Chen, Huawei Zheng, Lei Shi, Xu Zhao, Chengkang Zhang, Qingping Huang, Kunhai Fang, Guodong Lu, Daniel J. Ebbole, Guangpu Li, Naweed I. Naqvi, Zonghua Wang

**Affiliations:** 1 Fujian-Taiwan Joint Center for Ecological Control of Crop Pests, College of Life Science, Fujian Agriculture and Forestry University, Fuzhou, Fujian, China; 2 Fujian University Key Laboratory for Functional Genomics of Plant Fungal Pathogens, Fujian Agriculture and Forestry University, Fuzhou, Fujian, China; 3 Temasek Life Sciences Laboratory and Department of Biological Sciences, National University of Singapore, Singapore; 4 Department of Plant Pathology and Microbiology, Texas A&M University, College Station, Texas, United States of America; 5 Department of Biochemistry and Molecular Biology, University of Oklahoma Health Sciences Center, Oklahoma City, Oklahoma, United States of America; Purdue University, UNITED STATES

## Abstract

The retromer mediates protein trafficking through recycling cargo from endosomes to the trans-Golgi network in eukaryotes. However, the role of such trafficking events during pathogen-host interaction remains unclear. Here, we report that the cargo-recognition complex (MoVps35, MoVps26 and MoVps29) of the retromer is essential for appressorium-mediated host penetration by *Magnaporthe oryzae*, the causal pathogen of the blast disease in rice. Loss of retromer function blocked glycogen distribution and turnover of lipid bodies, delayed nuclear degeneration and reduced turgor during appressorial development. Cytological observation revealed dynamic MoVps35-GFP foci co-localized with autophagy-related protein RFP-MoAtg8 at the periphery of autolysosomes. Furthermore, RFP-MoAtg8 interacted with MoVps35-GFP *in vivo*, RFP-MoAtg8 was mislocalized to the vacuole and failed to recycle from the autolysosome in the absence of the retromer function, leading to impaired biogenesis of autophagosomes. We therefore conclude that retromer is essential for autophagy-dependent plant infection by the rice blast fungus.

## Author Summary

The rice blast fungus *Magnaporthe oryzae* utilizes key infection structures, called appressoria, elaborated at the tips of the conidial germ tubes to gain entry into the host tissue. Development of the appressorium is accompanied with autophagy in the conidium leading to programmed cell death. This work highlights the significance of the Vps35/retromer membrane-trafficking machinery in the regulation of autophagy during appressorium-mediated host penetration, and thus sheds light on a novel molecular mechanism underlying autophagy-based membrane trafficking events during pathogen-host interaction in rice blast disease. Our findings provide the first genetic evidence that the retromer controls the initiation of autophagy in filamentous fungi.

## Introduction

Rice blast is one of the most serious and recurrent diseases destroying rice production worldwide [1,2]. The ascomycete fungus *Magnaporthe oryzae* infects rice tissues by forming a dome-shaped and melanized infection structure called appressorium [3,4]. Differentiation of appressorium is regulated by cell cycle progression that is accompanied by autophagy in the conidium leading to its programmed cell death [5,6]. In this process, most of the stored glycogen and lipids are quickly transported from the conidium into the developing appressorium in order to establish a high turgor pressure necessary for successful host penetration by the mature appressorium [7–9]. Subsequently, a narrow penetration peg emerges from the mature appressorium and enters the rice epidermis and differentiates into bulbous, branched invasive hyphae, which are bound by the host plasma membrane in the invaginated cell, allowing the fungus to proliferate within the living plant cells [4,10].

Macroautophagy is a highly conserved bulk degradation process required for stress response and nutrient signaling in eukaryotes. Autophagy requires the formation of double-membrane bound autophagosomes that engulf bulk cytoplasm (nonselective) or specific target cargos (selective autophagy). The autophagosomes fuse with endosomes or the vacuoles to form autophagolysosomes to deliver the sequestered material for recycling and/or degradation [11,12]. A set of evolutionarily conserved autophagy-related genes (ATG genes) was initially identified in yeast [13,14]. In total, 22 ATG genes were identified in the rice blast fungus, and *MoATG8* expression has been used to investigate the spatial pattern of autophagy induction during infection-related development [5]. Punctate autophagosomes are found to be enriched in infection-related structures such as conidia, germ tubes and appressoria [15,16]. Deletion of *MoATG8* led to significant reduction in conidiation and defects in glycogen autophagy during conidiogenesis [15,16]. Furthermore, the *MoATG8*-deficient appressoria are nonfunctional and noninfectious due to an inability to undergo autophagic cell death and nuclear degeneration in conidia [5]. Genome-wide characterization of autophagy genes [15] further supports the critical role of autophagy in conidial cell death and the function of the appressorium in *M*. *oryzae*. However, the mechanisms regulating autophagy in *M*. *oryzae* remain elusive.

The retromer complex is a conserved vital element of the endosomal protein sorting machinery [17]. It consists of two subcomplexes: a trimer of Vps35, Vps29 and Vps26 for cargo selection, and a dimer of Vps5 and Vps17 for tubule or vesicle formation [18]. The retromer complex is known to participate in intracellular retrograde transport of cargos from the endosome to the proper organelles [19,20]. Loss or malfunction of retromer is associated with various pathological states due to protein mistargeting [17]. Serving as the core of retromer, Vps35 directly interacts with cargo proteins for sorting [19,20]. Recently, it was found that retromer played a role in the degradation of autophagic cargo to maintain lysosome function in Drosophila [21]. To our knowledge, the role of Vps35 or retromer in regulating autophagy and plant infection in plant fungal pathogens has not been assessed thus far.

In this study, we report a crucial role for the retromer cargo recognition subcomplex in regulating the autophagic process during appressorial development and pathogenesis in the rice blast fungus. We demonstrate that loss of any of these retromer components, MoVps35, MoVps26, or MoVps29, led to similar defects in fungal conidiogenesis and pathogenesis, which phenocopied the defects of ATG mutants including reduced turgor pressure, delayed turnover of glycogen and lipid bodies, and failure in autophagic cell death during conidial germination, and compromised pathogenicity of the blast fungus. Furthermore, our data suggest that MoVps35 regulates the autophagic process through retrieving the cleaved form of MoAtg8 from the vacuole after autolysosome formation. Therefore, our findings uncover a new function of retromer and shed light on the regulation of autophagy biogenesis in one of the most important fungal pathogens of rice and cereal crops.

## Results

### Generation and characterization of *Δ* *Movps35* deletion mutants

We identified a single Vps35 ortholog MoVps35 in the *M*. *oryzae* proteome using BLASTP analysis. MGG_05089 (hereafter MoVps35) showed 57% sequence identity to the yeast Vps35 (S1 Table). To determine its function, two *MoVPS35* null mutants were generated through targeted gene replacement with the hygromycin resistance cassette in the *Δku70* background (S1A and S1B Fig). Phenotypic analyses revealed that the mutant *ΔMovps35* grew marginally slower (about 72.4% of the WT, P<0.01) than the wild type on various culture media (S1C Fig). This suggests that the loss of *MoVPS35* likely reduces vegetative growth and/or overall fitness of *M*. *oryzae*. Given an important role for the cell wall in maintaining hyphal development and adaptation to the environment [22], we further investigated the growth under cell wall or membrane stress conditions. In such growth assays, the *ΔMovps35* showed decreased resistance to Calcofluor White (CFW), congo red (CR) and sodium dodecyl sulfate (SDS) compared to the wild-type strain (S2 Fig), suggesting that MoVps35 is involved in maintaining the integrity of the cell wall. We also found that in comparison to the wild type, *ΔMovps35* showed a 19-fold decrease in conidiation (S1D Fig and S1E Fig; P<0.01). The differentiation of conidiophores is critical for conidial development [23,24]. Although the sympodial arrangement of the resultant mutant conidia remained unchanged (S1D Fig), conidiophore differentiation was highly reduced in *ΔMovps35* at 24 h post conidial induction (S1F Fi.). Thus, the dramatic reduction of conidiogenesis in the *ΔMovps35* likely results from decreased conidiophore formation. Genetic complementation via introduction of *MoVPS35* restored proper growth and conidiation in the *ΔMovps35* strain (S1 Fig and S2 Fig). We conclude that MoVps35, and by inference the retromer function, is essential for proper vegetative growth and asexual development in the blast fungus.

### MoVps35 is essential for plant infection

**Fig 1:**
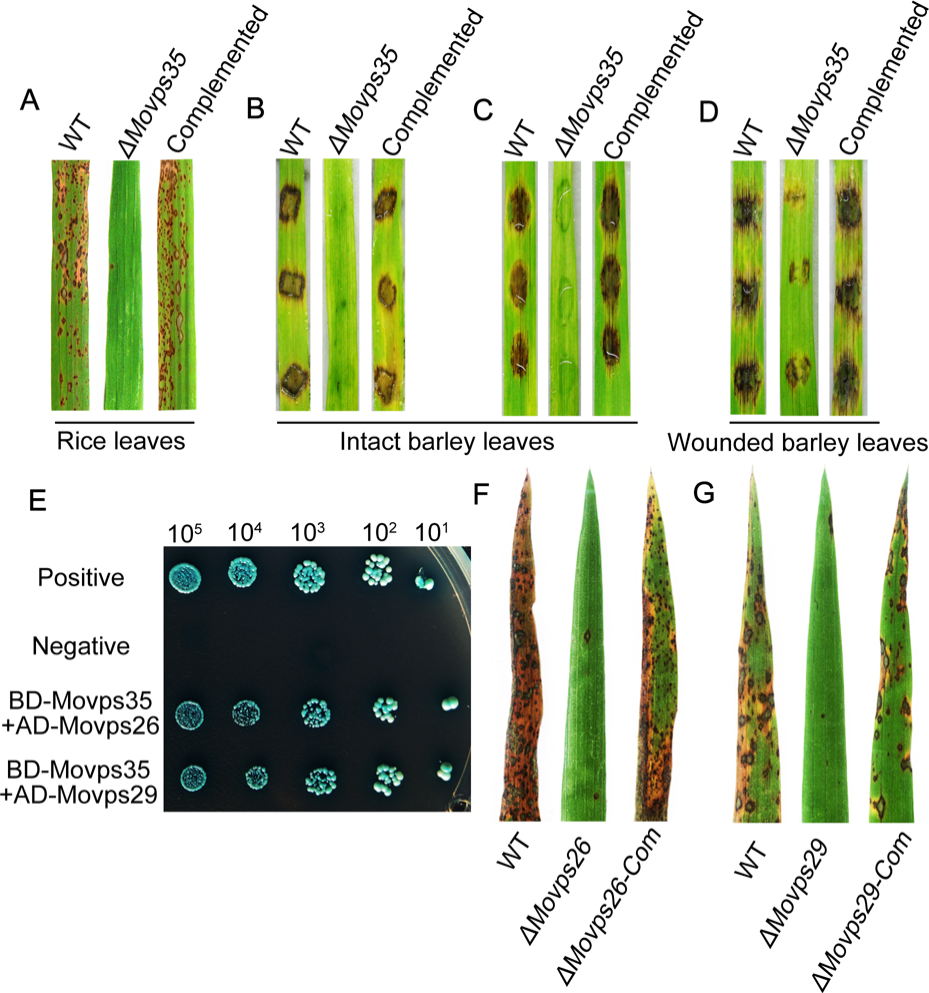
The retromer subcomplex MoVps35-MoVps26-MoVps29 is required for proper pathogenesis in the rice-blast fungus. (A) Pathogenicity assays on rice seedlings. Rice leaves (*Oryza sativa* cv. CO39) sprayed with conidia from the wild-type strain *ΔKu70*, *ΔMovps35* mutant and the complemented *ΔMovps35* strain. (B), (C), (D) are pathogenicity assays on barley leaves. Barley leaves were inoculated with mycelial plugs (B) or with 20 microliter of conidial suspension (ca 10^5^ conidia/mL) (C). (D) Barley infection using wounded/abraded leaves. Lesion formation on leaves was observed 7 days after inoculation with mycelial plugs from the indicated strains. (E) Yeast two-hybrid assay for the interaction between retromer components. (F) and (G) Mutant analysis in *MoVPS26* and *MoVPS29* suggests a role for cargo-specific retromer in *M*. *oryzae* pathogenicity. Conidia from the indicated strains were inoculated on the susceptible rice (CO39) seedlings and imaged 7 days post inoculation.

We then assessed the pathogenicity of *ΔMovps35* mutant on rice seedlings (*Oryza sativa* cv. CO39). When spray-inoculated on rice seedlings, the wild type as well as the complementation strain caused numerous typical blast lesions on leaves, whereas the *ΔMovps35* mutant caused only a few small and isolated lesions (Fig 1A). *ΔMovps35* formed only 1.2 ± 1 lesions per 5 cm of leaf (P<0.01), whereas 69.7 ± 15.3 lesions were evident in leaves inoculated with wild-type conidia (Fig 1A). Likewise, the barley infection assays (cv. Golden Promise) showed severe blast symptoms seven days after inoculation with wild-type conidial suspension or mycelia, whereas the *ΔMovps35* mutant failed to cause blast disease in barley seedlings (Fig 1B and Fig 1C). To investigate if the pathogenicity defects in *ΔMovps35* were due to a block in penetration or invasive growth, we inoculated mycelia from the wild type or *ΔMovps35* through abraded barley leaves. This allows for invasive growth independent of appressorium function. We found that the *ΔMovps35* mycelia were able to invade the wounded tissue and caused weak lesions on the wounded leaves compared to the WT (Fig 1D). These results suggested that the *ΔMovps35* mutant were unable to infect rice and barley, owing mainly to their inability to penetrate the plant cuticle.

### MoVps26 and MoVps29, are also required for the initiation of blast disease

In yeast, plant and mammals, Vps26p and Vps29p form the cargo-selective subcomplex of the retromer via interaction with Vps35p [25,26]. Therefore, we assessed whether these proteins cooperate in common pathogenic pathways in *M*. *oryzae*. Using orthologous yeast sequences, we identified MGG_04830 and MGG_02524 as MoVps26 and MoVps29, respectively, in *M*. *oryzae* (S1 Table). We first investigated whether such a retromer subcomplex occurs in *M*. *oryzae*. In the yeast two-hybrid assay, MoVps35 was found to physically interact with MoVps26 and MoVps29 (Fig 1E). Furthermore, we constructed *ΔMovps26* and *ΔMovps29* deletion mutants by gene replacement. The *ΔMovps26* and *ΔMovps29* deletion mutants (S2 Table) were identified by PCR and confirmed by DNA gel blot analysis (S3 Fig). Like the *ΔMovps35* mutant, *ΔMovps26* and *ΔMovps29* mutants were also impaired in conidiation and pathogenicity on the seedlings of the susceptible rice cultivar CO39 (S4 Fig, Fig 1F and Fig 1G). Finally, we expressed WT *MoVPS26* and *MoVPS29* gene in the corresponding null mutants. As expected, the complementation strains showed suppression of mutant defects in conidiation and pathogenicity (S4 Fig, Fig 1F and Fig 1G). The data suggest that MoVps35, MoVps26 and MoVps29 function together in the retromer pathway and play a key role in plant infection by the rice blast fungus.

### The MoVps35 is essential for appressorium-mediated host penetration

**Fig 2:**
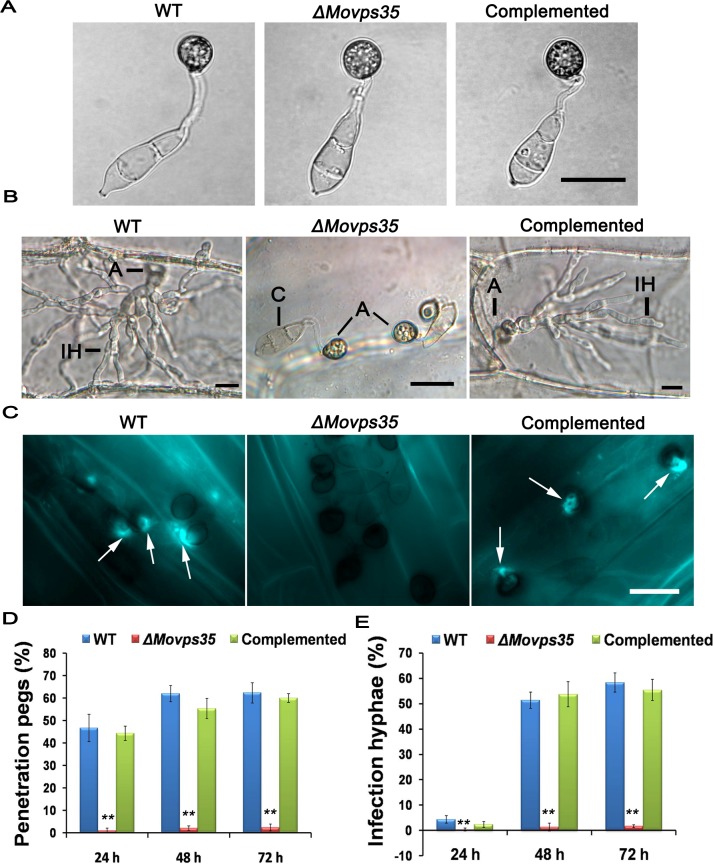
MoVps35 is essential for host penetration during rice blast. (A) Infection-related morphogenesis assays. Normal morphology and melanized appressoria formed by the wild type, *ΔMovps35* mutant and complemented strains on plastic cover slips. (B) Penetration assays with onion epidermal cells. By 48 h after inoculation, invasive hyphae were evident in plant cells penetrated by WT and complemented strains but not by the *ΔMovps35* mutant. A, appressorium; C, conidium; IH, invasive hyphae. (C) Equal number of conidia from WT, *ΔMovps35* and complemented strains were inoculated on barley leaves. At 48 hpi, the papillary callose deposits (arrowheads) were evident in the barley leaves inoculated with conidia from the WT or complemented strains, but were absent in *ΔMovps35*-inoculated samples. Scale Bars = 20 μm. (D) and (E) Quantifications of penetration pegs and infection hyphae from three independent experiments at each time point, where values are indicated as percentages. The double asterisks indicate statistically significant differences (P < 0.01).

To understand why the retromer subcomplex (MoVps35, MoVps26 and MoVps29) is required for pathogenicity in *M*. *oryzae*, we first chose the core retromer component MoVps35 for a detailed functional analysis. Based on the above observations, we reasoned that MoVps35 either controls proper appressorium formation or appressorium-mediated infection in *M*. *oryzae*. We first assayed for appressorium formation on artificial hydrophobic surfaces, wherein the *ΔMovps35* conidia produced abundant melanized appressoria (Fig 2A). No obvious morphological defects in appressorium formation were evident in *ΔMovps35* (Fig 2A–2C). The *ΔMovps35* exhibited normal appressorium formation on onion epidermal cells as well (Fig 2B). However, such *ΔMovps35* appressoria were defective in the penetration of onion epidermal cells and subsequent differentiation into invasive hyphae (Fig 2B). By 48 h, the wild-type strain penetrated and formed invasive hyphae in onion epidermal cells (Fig 2B). A vast majority of the appressoria (95%, P<0.01) failed to penetrate the onion epidermal cells even at 72 hpi. Aniline blue staining and further quantification of penetration efficiency was carried out on barley leaves inoculated with conidia of WT or *ΔMovps35* (Fig 2C). The efficiency to form penetration pegs on barley leaves was about 62% in WT, while only 3.6% (P<0.01) appressoria showed host entry in the *ΔMovps35* mutant at 48 h time point. Even upon extended incubation, *ΔMovps35* appressoria were still unable to penetrate the host surface (Fig 2D and 2H). Similar defects were also evident in invasive growth in *ΔMovps35* inoculated on barley leaves (Fig 2E). We conclude that MoVps35 is not required for appressorium formation but is essential for appressorium-mediated host penetration by the rice blast fungus.

### *ΔMovps35* fails to establish full turgor pressure in appressoria

**Fig 3:**
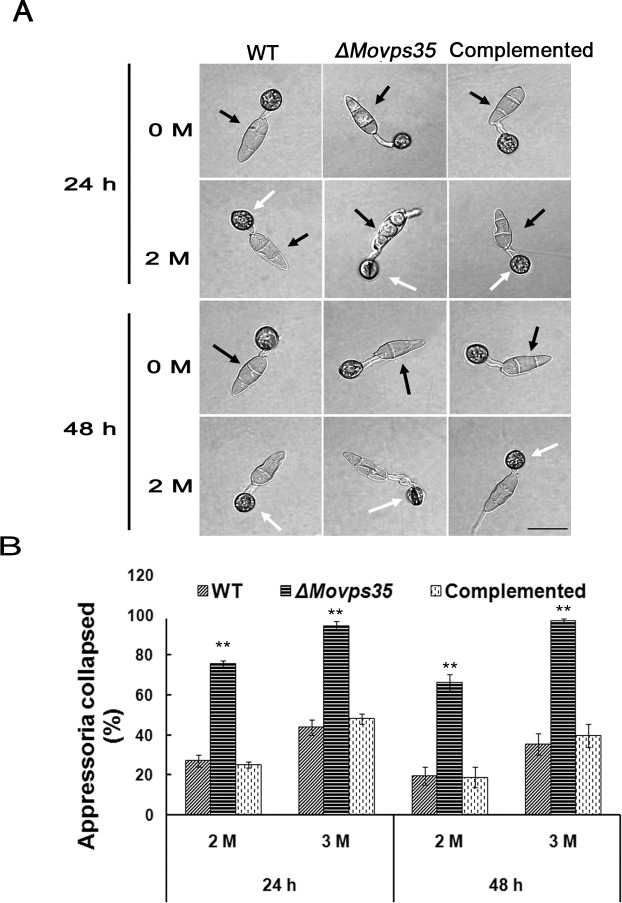
MoVps35 regulates appressorium turgor generation. (A) Appressorium turgor was measured by incipient cytorrhysis assays. Appressoria were allowed to form on plastic coverslips for 24 h or 48 h, and the collapsed appressoria assessed after exposure to 2 M glycerol solutions. White arrows indicate the appressoria in the wild type (WT) or *ΔMovps35* strain. Black arrows indicate the conidial morphology when appressoria formed after 24 h or 48 h. Wild-type strain showed typical collapsed conidia during appressorial maturation (24 hpi). However, the conidia from the *ΔMovps35* mutant were still intact and turgid. Bar = 20 μm. (B) Proportion of collapsed appressoria after exposure of conidia to 2 M or 3 M glycerol, double asterisks indicate statistically significant differences (P < 0.01).

Since establishment and maintenance of high internal turgor pressure is necessary for appressorium-mediated host penetration by *M*. *oryzae* [9], we examined the turgor pressure in *ΔMovps35* using the incipient cytorrhysis assays [27]. These appressorial collapse assays revealed that the *ΔMovps35* appressoria generate significantly lower turgor compared to wild type (Fig 3; P<0.01). In 2 M glycerol, 75% of *ΔMovps35* appressoria collapsed at 24 h compared to 27% and 25% of the appressoria in the wild type and complementation strains, respectively (Fig 3B). Upon increasing the glycerol to 3 M and extending the incubation, appressoria of *ΔMovps35* mutant remained severely collapsed compared to those from the wild-type or the complemented strain (Fig 3B). Further analysis revealed that the wild-type strain completely transferred the cytoplasm from conidia into the appressoria, leading to collapsed conidial morphology, while a large proportion of the cytoplasm was still intact in the mutant cells and consequently the conidia remained intact and turgid at 24 h after incubation (Fig 3A; black arrows).

### Mobilization of glycogen and lipid bodies from conidia to appressoria is blocked in the *ΔMovps35* mutant

**Fig 4:**
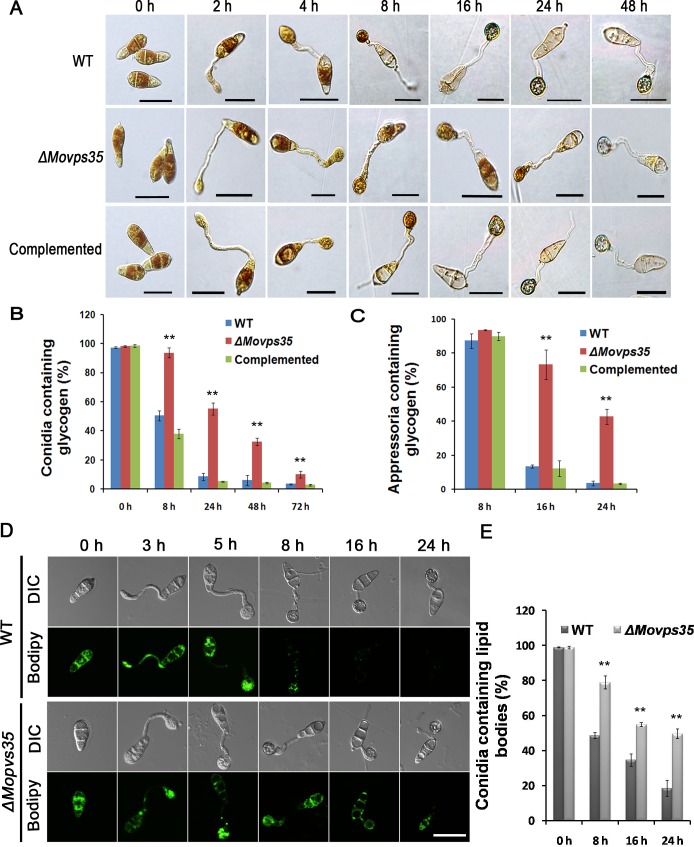
Cellular distribution of glycogen and lipid bodies during conidial germination and appressorium morphogenesis. (A) Conidia from wild type, *ΔMovps35* and the complemented strain were germinated on hydrophobic gelbond membranes. Drop of water was replaced by iodine solution at 0 h, 2 h, 4 h, 8 h, 16 h, 24 h and 48 h to stain for 2 minutes and microscopically visualize glycogen as yellowish-brown deposits. The *ΔMovps35* mutant is impaired in turnover/mobilization of glycogen from the conidium to appressorium and in subsequent degradation within the appressorium. (B-C) Quantitation of the total glycogen content in conidia and/or appressoria during pathogenic development in the indicated strains. Assays from three independent experiments in each time point, where values are indicated as percentages. The double asterisks indicate statistically significant differences (P < 0.01). (D) MoVps35 is involved in lipid body translocation and degradation during appressorium morphogenesis. Conidia of *M*. *oryzae* WT, and *ΔMovps35* were incubated in water droplets on the hydrophobic surface of gelbond and allowed to form appressoria for up to 24 h. Samples were removed at 0, 3, 5, 8, 16 and 24 h and stained with Bodipy to observe the presence of lipid bodies by confocal microscopy. (E) Quantitative analysis of lipid bodies during appressorium morphogenesis. The bar charts show the mean and standard deviation from three independent replicates of the experiment. Values shown with double asterisks are statistically significant at P < 0.01. Bars = 20 μm.

The delayed mobilization of cytoplasmic content into the appressoria prompted us to further investigate the germinating conidia. In *M*. *oryzae*, conidia contain several sources of stored energy such as glycogen and lipids [7], and effective transfer of such materials is required for appressorial maturation and appressorium-mediated host penetration [7,28]. We therefore examined the cellular distribution of glycogen and lipid bodies during appressorium development. Upon iodine-staining abundant glycogen was seen in conidia, germ tubes, and incipient appressoria of the WT and *ΔMovps35* from 0 h to 4 h during germination of conidia on a hydrophobic surface (Fig 4A). However, mobilization of glycogen was notably retarded in *ΔMovps35* mutant after 8 h conidial germination (Fig 4A). Even after 24 h, a significantly higher proportion of mutant conidia contained glycogen (Fig 4B). Therefore, glycogen catabolism/hydrolysis was greatly delayed in the *ΔMovps35* mutant (Fig 4A–4C). Next, we investigated the distribution of lipid bodies by Bodipy staining and confocal microscopy. The distribution of lipid bodies showed the same pattern as glycogen in the *ΔMovps35* mutant, as shown in Fig 4D and Fig 4E. The data indicate that the mobilization of glycogen and lipid bodies from conidia to the appressoria is greatly reduced or blocked in the *ΔMovps35* mutant.

Like MoVps35, the other two components, MoVps26 and MoVps29, of the retromer subcomplex were also found to be necessary for proper initiation of blast disease, as judged by the similar phenotypic defects in glycogen distribution, lipid droplet turnover, and appressorial turgor generation shown by the requisite *ΔMovps26* and *ΔMovps29* (S5 Fig). Taken together, these results indicate that the cargo-recognition subcomplex of the retromer comprising of MoVps35, MoVps26 and MoVps29, functions in mediating critical physiological/metabolic processes associated with pathogenic differentiation in *M*. *oryzae*.

### *MoVPS35* deletion impairs conidial autophagic cell death and biogenesis of autophagosomes

**Fig 5:**
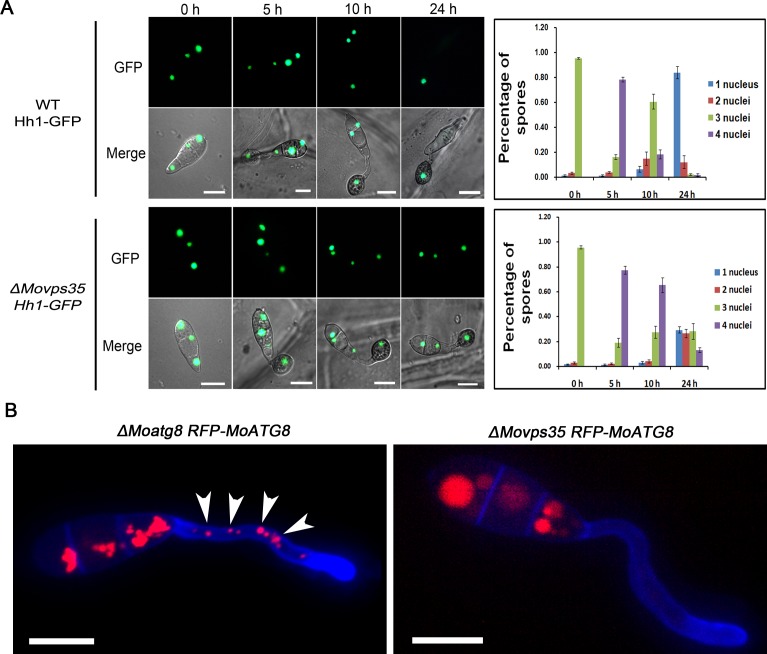
*MoVPS35* is required for nuclear degeneration in conidia and biogenesis of autophagosome during appressoria maturation. (A) Micrographs showing delayed nuclear degeneration in *ΔMovps35* conidia during appressorium development. The bar charts on the right side show the percentage of spores in wild-type strain and *ΔMovps35* mutant containing 0 to 4 nuclei (n>100, triple replicates) during appressorium development. (B) Cellular localization of autophagosomes in conidial germination. RFP-MoAtg8 punctate structures (arrows) are absent in germ tubes of *ΔMovps35 RFP-MoATG8* when compared to *ΔMoatg8 RFP-MoATG8*. Bar = 10 μm.

Studies in *M*. *oryzae* have shown that proper conidiation and appressorium formation/function requires autophagy-assisted utilization of carbohydrate(s), glycogen or stored lipids [16,29,30]. Autophagy-deficient mutants (*Δatg1*, *Δatg2*, *Δatg4*, *Δatg5*, *Δatg8*, *Δatg9* and *Δatg18*) show delayed breakdown of glycogen and lipid bodies, reduced turgor pressure and complete loss of pathogenesis in *M*. *oryzae*. [29,31–35]. Similar defects in *ΔMovps35* prompted us to investigate whether MoVps35 is directly involved in regulating autophagy function(s) during appressorium-mediated host penetration. To test this idea, an Hh1-GFP (encodes nuclear localized Histone H1) was introduced into the wild type and *ΔMovps35* strains to allow live cell imaging of nuclear degeneration associated with autophagic cell death [5] (S2 Table). In WT, the number of Hh1-GFP marked nuclei gradually decreased due to autophagy-based degeneration in conidial cells during appressorial maturation. As a result, a single nucleus remains intact in the mature appressorium of the wild-type strain at 24 hpi (84%, P<0.01) (Fig 5A). However, the majority of the Hh1-GFP expressing *ΔMovps35* conidia (71%) contained more than one nucleus (Fig 5A). These data suggest that MoVps35 deficiency affects the autophagic cell death in conidia of *M*. *oryzae*.

A key event in autophagy is the formation of a double-membrane autophagosome, which engulfs portions of cytosol and entire organelles [36,37]. Thus, we sought to determine whether retromer plays a role in the formation of autophagosomes during appressorium development. We used the RFP-MoAtg8 as an epifluorescent marker for autophagosomes [16,38–40]. MoAtg8 is a ubiquitin-like protein that marks autophagosomal structures and is required for the formation of autophagosomes. RFP-MoAtg8 was expressed under the control of the endogenous *MoATG8* promoter in the *ΔMoatg8* or *ΔMovps35* background. Analysis of *ΔMoatg8 RFP-MoATG8* showed typical punctate autophagosomes and vacuolar autolysosomes widely distributed in conidia, germ tubes and appressoria (Figs 5B and S6). However, the *ΔMovps35 RFP-MoATG8* strain showed no obvious punctate autophagosomes in germ tubes and appressoria, except for aggregated red epifluorescence signal in the vacuoles (Figs 5B and S6). We reasoned that RFP-MoAtg8 was probably retained in the vacuole and subsequently degraded by the vacuolar hydrolase upon loss of MoVps35 function. The dynamics of autophagic structures was investigated using time-lapse microscopy. In *ΔMoatg8 RFP-MoATG8* strain, mobile spherical autophagosomes (about 1 μm diameter) fuse with vacuolar structures (2–5 μm diameter) and also dissociate from these structures (S1 Movie), indicating that autophagosomes cooperatively act to form autophagolysosomes or are recovered once autophagy is completed. By contrast, there are no obvious spherical autophagosomes in the *ΔMovps35 RFP-MoATG8* strain (S2 Movie). These results suggest that MoVps35 is necessary for the proper localization of MoAtg8 and consequently required for autophagosome formation during appressorial maturation.

### MoVps35, MoVps26 and MoVps29, are associated with the periphery of vacuoles/autolysosomes

**Fig 6:**
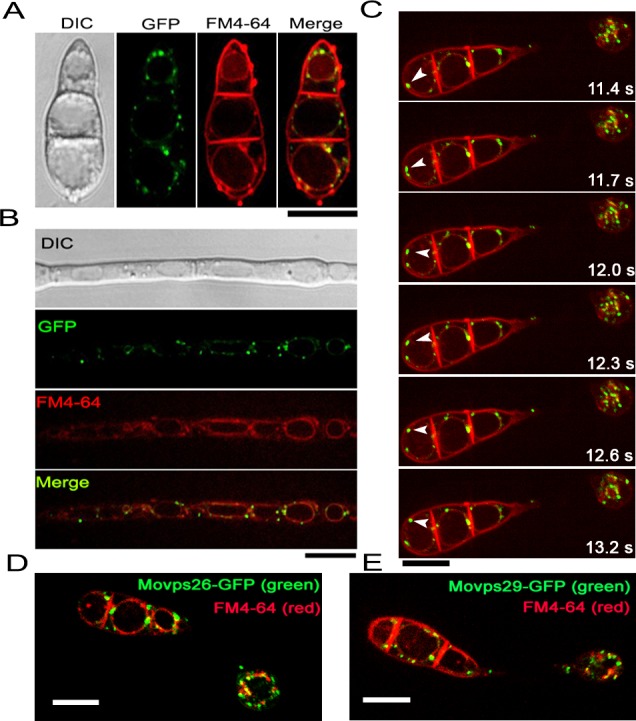
Subcellular localization of the retromer subcomplex. MoVps35-GFP localizes to punctate structures at or near the vacuolar membrane, and partially colocalizes with FM4-64 that marks endosomal and vacuolar membranes in conidia (A) and mycelium (B). Shown are confocal epifluorescent micrographs. Scale bar = 10 μm. (C) Time-lapse video microscopy of MoVps35-GFP-labeled punctate structures. Arrowheads indicate the relative position of MoVps35-GFP-labeled compartments at each time point. Elapsed time is indicated in seconds. See also S3 Movie. Scale bar = 10 μm. (D) and (E) show localization of MoVps26-GFP and MoVps29-GFP to similar punctate structures at or near the vacuolar membranes. Scale bars = 10 μm.

To investigate the mechanism by which retromer participates in the regulation of autophagosome formation, we monitored the dynamics of MoVps35 trafficking in *M*. *oryzae* using a *ΔMovps35 pMoVPS35*::*MoVPS35-GFP* strain (S2 Table). The *pMoVPS35*::*MoVPS35-GFP* construct complemented all the defects found in *ΔMovps35* mutants (Fig 1), indicating that MoVps35-GFP is fully functional. MoVps35-GFP exhibited a mostly punctate pattern at or near the vacuolar membrane in conidia and mycelia (Fig 6A and 6B). The association with vacuoles was confirmed by staining with the lipophilic styryl dye FM4-64 (Fig 6A and 6B). Furthermore, we investigated the spatiotemporal dynamics of MoVps35-GFP during infection-related development. MoVps35-GFP consistently localized to small punctate/vesicular compartments (approximately 0.5–2.0 μm) in conidia, germ tubes and nascent appressoria by 2 h and 4 h, respectively (S7 Fig). During 8–24 h, the fluorescent signal was predominant in developing appressoria and gradually diminished in the conidia, consistent with conidial autophagic cell death (S7 Fig). Next, we monitored the dynamics of MoVps35-GFP movement in conidia and appressoria during conidial germination upon staining with FM4-64. MoVps35-GFP was present on highly mobile punctate structures in germinated conidia and developing appressoria (Fig 6C; S3 Movie). Strikingly, the movement of MoVps35-GFP-containing structures was not random but inherently associated with vacuolar membranes or late endosomes as judged by FM4-64 staining (Fig 6C; S3 Movie). We interpret these epifluorescence traces of MoVps35-GFP as evidence for vesicular trafficking in the late endosomal compartments. Moreover, the mobility of MoVps35-GFP depends on microtubules but not the F-actin cytoskeleton, because it was disrupted by the microtubule-destabilizing agent MBC (Methyl Benzimidazol-2-yl-Carbamate) but not by actin-depolymerizing drug LatA (latrunculin A) (S8 Fig and S4–S6 Movies). In addition, like MoVps35-GFP localization, MoVps26-GFP and MoVps29-GFP both exhibited a similar dynamic and punctate pattern on the vacuolar membrane (Fig 6D and 6E). Spatiotemporal dynamic of MoVps26-GFP and MoVps29-GFP distribution during infection-related development was also reminiscent of the MoVps35-GFP localization (S9 Fig, S7 and S8 Movies). These data suggest that retromer may function in the retrieval of cargo associated with vacuoles or autolysosomes in *M*. *oryzae*.

### MoVps35 dynamically colocalizes and interacts with MoAtg8

**Fig 7:**
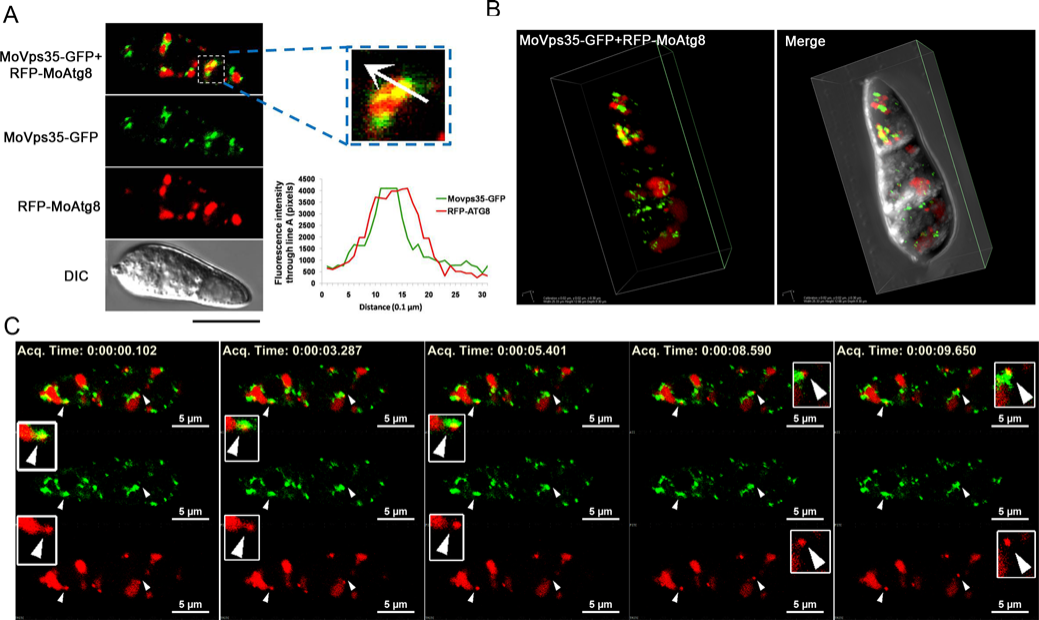
MoVps35 is required for retrieving MoAtg8. (A) Close association between MoVps35-GFP (green) and RFP-MoAtg8 (red) in conidia. The enlarged inset highlights compartments showing co-localized MoVps35-GFP and RFP-MoAtg8. White arrow in the inset shows the path for fluorescence intensity distribution by line-scan analysis. Bar = 10 μm. (B) 3D images showing the association of MoVps35-GFP with RFP-MoAtg8 in conidia. (C) Images of a time-lapse sequence from S10 Movie showing MoVps35-GFP-mediated retrieval of punctate RFP-MoAtg8. Arrowheads mark dynamic dissociation process of RFP-MoAtg8 and the colocalization between MoVps35-GFP and RFP-MoAtg8. The boxed regions (in white) highlight compartments showing dynamic retrieval of RFP-MoAtg8 by MoVps35-GFP. From top to bottom are images of MoVps35-GFP and RFP-MoAtg8 (merged), MoVps35-GFP (alone), and RFP-MoAtg8 (alone), Bars = 5 μm. Elapsed time is indicated in millisecond.

Given the essential role of retromer in the formation of autophagosomes and the apparent association with late endosomes, we suspected that MoVps35-GFP motility might contribute to the retrieval of MoAtg8 to pre-autophagosomal structures and autophagosomes. To test this hypothesis we first determined whether MoVps35 colocalizes with MoAtg8. *pMoVps35*::*MoVPS35-GFP* was introduced into the *ΔMoatg8 RFP-MoATG8* strain (S2 Table) for localization and dynamic association analysis. In fresh harvested conidia, most of MoVps35-GFP vesicles were arranged adjacent to RFP-MoAtg8 labeled organelles, implying a potential association between these two compartments (Fig 7). Remarkably, a proportion of MoVps35-GFP punctae colocalized with the cytosolic RFP-MoAtg8 compartments as determined by line-scan and 3D reconstruction analysis (Fig 7A and 7B, see also S9 Movie). In order to test whether the colocalization existed during other developmental stages of *M*. *oryzae*, conidia from the dual-labeled *ΔMoatg8 RFP-MoATG8 MoVPS35-GFP* strain were incubated in vitro to observe germination and appressoria formation using confocal microscopy. At 2 h, many germ tubes initiated appressorium formation. In addition to the partially colocalized/overlapping RFP and GFP fluorescent signals detected in conidia, a small proportion of such colocalized signals were also apparent in the germ tubes (S10A Fig). A similar localization pattern was also found in developing appressoria (S10B Fig). Furthermore, RFP-MoAtg8 partially co-localized with MoVps35-GFP in vegetative hyphae under nitrogen starvation conditions that induce autophagy (S10C Fig). In order to directly record spatial and temporal association between MoVps35-GFP and RFP-MoAtg8, a real time imaging was applied. S10 Movie or time-lapse Fig 7C shows a conidium undergoing autophagy, RFP-MoAtg8 fluorescent were highly associated with oblong vacuoles (approximately 2–5 μm diameter) and spherical structures (approximately 1 μm diameter). Interestingly, we observed that mobile RFP-MoAtg8 puncta showed a rapid dissociation from the adjacent vacuoles, and at the same time the MoVps35-GFP also displayed very close colocalization with the punctate RFP-MoAtg8 (Fig 7C, arrows). This suggests that MoVps35 might play an important role for retrieving MoAtg8 from the vacuole, avoiding unnecessary degradation by vacuolar hydrolases.

**Fig 8:**
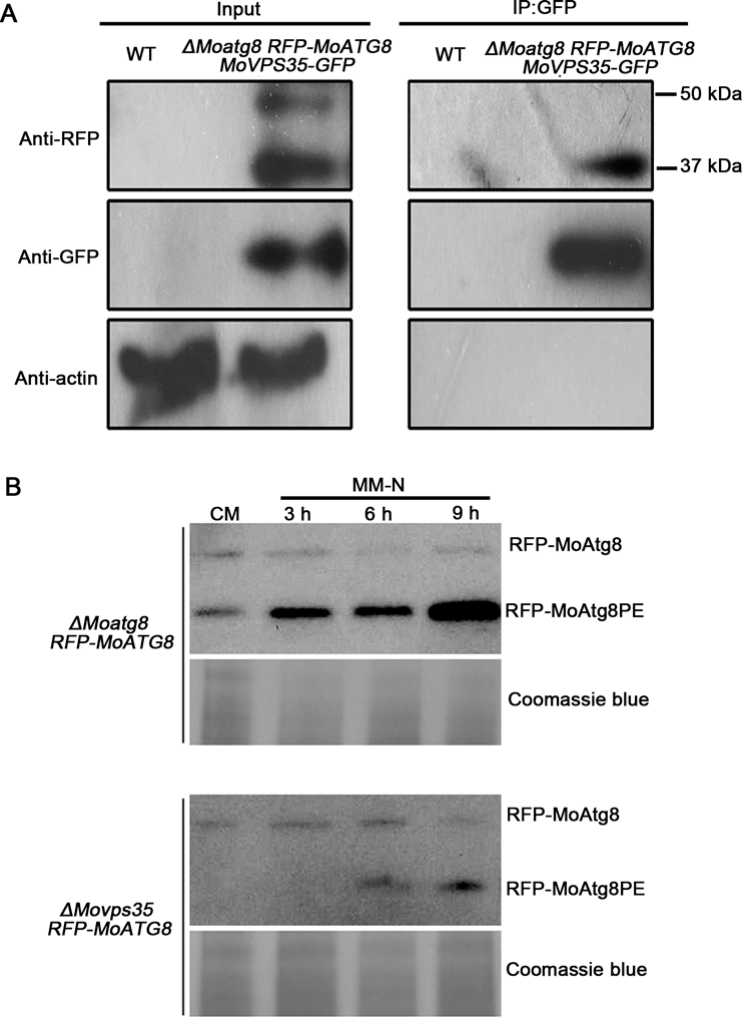
MoVps35 interacts with MoAtg8 and is involved in the retrieval of MoAtg8. (A) GFP-trap based pull down experiment indicating the interaction between MoVps35-GFP and RFP-MoAtg8 in the transformant. Total proteins isolated from the wild-type strain (WT) are included as a negative control. MoVps35-GFP shows specific interaction with the cleaved and lipidated MoAtg8 during MM-N induced starvation. Top, middle, and bottom images represent immunoblot detection with anti-RFP, anti-GFP, and anti-actin antibodies, respectively, as indicated. (B) Delayed post-translational processing of MoAtg8 in *ΔMovps35* mutant. Immunoblot analysis of total lysates from CM and MM-N cultured (3 h to 9 h in starvation environment) *ΔMoatg8 RFP-MoATG8* and *ΔMovps35 RFP-MoATG8* transformants, with anti-RFP antibody. Coomassie blue staining of total lysates serves as a loading control.

To test whether MoVps35 contributes to the retrieval of MoAtg8 *in vivo*, we applied a GFP-trap/co-immunoprecipitation assay to pull down MoVps35-GFP and found that anti-RFP antibody was able to specifically detect a clear band of about 37 kD, the size of the truncated variant of RFP-MoAtg8 (Fig 8A). No unmodified full-length RFP-MoAtg8 band was detected from the proteins pulled down with MoVps35-GFP (Fig 8A). In the control experiment, both truncated RFP-MoAtg8 variant (approximately 37 kD) and full-length RFP-MoAtg8 (approximately 48 kD) were detected with an anti-RFP antibody with input protein isolated from the *ΔMoatg8 RFP-MoATG8 MoVPS35-GFP* strain (Fig 8A). These results indicate that MoVps35 is able to specifically interact with the truncated variant of MoAtg8, which is consistent with the time-lapse microscopy results of MoVps35-GFP and RFP-MoAtg8 in *M*. *oryzae*. Taken together, the MoVps35 acts through a direct interaction with truncated variant of MoAtg8 and contributes to its retrograde transport in *M*.*oryzae*.

Autophagy can be measured by examining the intracellular levels of cleaved and lipidated Atg8, viz Atg8PE, which is the key protein known to associate specifically with autophagosomes, as its levels correlate with the number of autophagosomes [38–40]. If MoVps35 is required for the retrieval of MoAtg8 to phagophore assembly sites (PAS)/autophagosomes, we would anticipate that the level of cleaved and lipidated MoAtg8 (ie., MoAtg8PE) would be reduced in *ΔMovps35* under the conditions that induce autophagy. Therefore, we further used immunoblot assays to analyze the levels of the cleaved MoAtg8 protein in *ΔMoatg8* and *ΔMovps35* strains expressing *RFP-MoATG8* (Fig 8B). We detected two forms of RFP-MoAtg8 using anti-RFP antibody, inferring that these represented the full-length and lipidated form, MoAtg8PE. Under nitrogen starvation condition, the amount of RFP-MoAtg8PE gradually increased in the *ΔMoatg8 RFP-MoATG8*, which correlated well with the extent of autophagosome formation (Fig 8B). However, in the *ΔMovps35 RFP-MoATG8* strain, the levels of RFP-MoAtg8PE were greatly reduced and delayed in both CM and MM-N medium compared to the *ΔMoatg8 RFP-MoATG8* strain, indicative of fewer autophagosomes (Fig 8B). Interestingly, the autophagy flux was not completely blocked in the *ΔMovps35* since some RFP-MoAtg8PE still accumulated in the cells upon nitrogen starvation (Fig 8B). These findings are consistent with the microscopy results of scarce spherical autophagosomes in the *ΔMovps35 RFP-MoATG8* strain.

Besides, the compromised expression levels of *MoATG8* in the *ΔMovps35* mutant could also lead to the lack of MoAtg8-marked autophagosome. Therefore, we further used qRT-PCR to assess the expression levels of the *MoATG8* gene in *ΔMovps35* and wild-type strains under the MM-N conditions. Compared to the wild-type strain, the expression levels of *MoATG8* do not differ significantly in the *ΔMovps35*, but appear to be mildly upregulated (S11A Fig). We further analysed the expression levels of *MoATG4* which is a key cysteine protease responsible for the cleavage of the carboxy terminus of MoAtg8 during the biogenesis of autophagosomes in *M*. *oryzae* [31]. Similar to *MoATG8*, the expression of *MoATG4* was slightly higher in the *ΔMovps35* as compared to the wild type (S11A Fig), suggesting that MoVps35 regulates the biogenesis of punctate autophagosomes primarily via modulating the retrieval of MoAtg8, but not by compromising the expression of *MoATG8* and *MoATG4* in *M*. *oryzae*. These results also explain why increased expression of *MoATG8* is unable to rescue the phenotypic defects in *ΔMovps35* mutant (S1 11B–S11F Fig).

**Fig 9:**
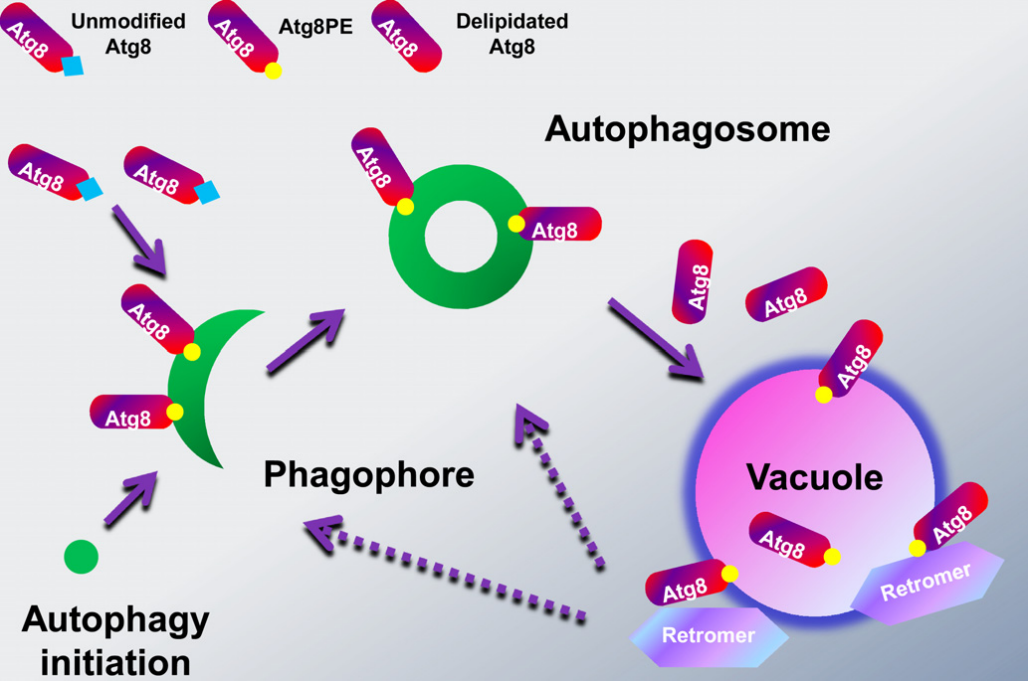
A model for the retromer function in Atg8 retrieval. Autophagy requires the formation of double-membrane bound autophagosomes that associate with a set of evolutionarily conserved autophagy-related proteins, including Atg8. During the autophagocytosis, Atg8 has to be conjugated to the lipid phosphatidylethanolamine (PE), resulting in the expansion of the phagophore membrane and formation of autophgosomes. At the late stage, the autophagosomes fuse with the vacuoles to form autophagolysosomes to deliver the sequestered material for degradation and/or recycling. In yeast, the outer membrane-bound Atg8 was released into the cytosol by delipidation before autophagomes-vacuole fusion, presumably for reuse in subsequent rounds of autophagosome formation. In our study, we found that *M*. *oryzae* retromer core complex (MoVps35-MoVps26-MoVps29) localized at the late endosome/ prevacuolar membranes, where it interacts with cleaved and lipidated Atg8 thus regulating its trafficking to the phagophore or autophagosome and preventing its degradation in the lumen of vacuole.

Overall, the retrograde transport function of retromer complex, the close association between retromer core component MoVps35 and the key autophagy protein MoAtg8, and their tight functional link in autophagocytosis, asexual differentiation and plant infection provides an insight into a novel function of regulating the biogenesis of autophagosomes by retrieving cleaved MoAtg8 from the vacuolar compartments for targeting to the proper structures in the rice blast fungus (Fig 9).

## Discussion

In this work, we have addressed the outstanding question about the mechanism of Atg8 retrieval during autophagosome biogenesis. It was previously reported that the majority of Atg8 molecules were released into the cytoplasm before autophagosome–vacuole fusion, suggesting that Atg8 is retrieved for the formation of autophagosomes [41,42]. However, the molecular mechanisms for Atg8 retrieval remained unclear. Our results show that MoVps35, a retromer core component that functions in endosomal sorting, directly interacts with MoAtg8 and is associated with MoAtg8 retrieval process from the periphery of vacuoles (Fig 9). Loss of retromer function leads to the mistargeting of RFP-MoAtg8 to the vacuole and thus the impairment of the biogenesis of autophagosomes. Moreover, the phenotype of all retromer component mutants mimic the morphological disturbance observed with *ΔMoatg8*, including failure to undergo autophagic cell death during conidial germination, and defects in fungal appressorium-mediated pathogenesis. Taken together, our results provide the first clear linkage between the retrograde transport mediated by retromer complex and the autophagy-dependent plant infection.

The highly conserved retromer is well known to function in the retrieval of recycling cargos to TGN in the retrograde pathway, but its unconventional roles are now beginning to emerge. In *Arabidopsis thalliana*, the retromer complex components Vps35, Vps26 and Vps29 localize to the prevacuolar compartment (PVC) and are essential for normal PVC morphology [43,44]. Mutations in *VPS35* or *VPS29* in *A*. *thalliana* lead to a dwarf phenotype and defects in PIN protein repolarization, embryogenesis, plant growth, and leaf senescence [45,46]. In *Drosophila melanogaster*, Vps35 function is necessary for normal endocytic trafficking and organization of the F-actin cytoskeleton [47]. Loss of Vps35 severely affects endocytosis and the localization of a number of endocytic proteins, causes defects in signaling pathways in haemocytes and at the neuromuscular junction, and leads to increased levels of F-actin [47]. Interestingly, a recent report found that the mammalian retromer regulates trafficking and subsequent incorporation of HIV-1 envelope glycoprotein (Env) into virions [48]. Inactivating retromer alters Env localization, cell surface expression and incorporation into virions, and the binding of retromer to the Env cytoplasmic tail is required for these functions [48]. Our study suggests an important role in retromer is essential for the autophagy-dependent plant infection in *M*. *oryzae*, thus expanding the functions of retromer. Interestingly, although the retromer is important for appressorium-mediated host penetration, it is not essential for colonization therein. This suggests that the retromer and efficient autophagy is not required for suppression of host defense, invasive growth within the rice cells, or spread from cell-to-cell in the host. It will be interesting to investigate whether the retromer plays a similar role in autophagy and infection-related development in other fungus-plant pathosystems.

Our cell biological, biochemical and genetic analyses demonstrate that the retromer is essential for autophagy by activating the formation of autophagosomes through the Vps35’s direct interaction with and retrieval of MoAtg8 in the *M*. *oryzae*. This role of the retromer could be widespread throughout the eukaryotic kingdom. Recently, two studies have implicated a role for the retromer in autophagy in yeast and mammalian cells. Dengjel and his colleagues used quantitative proteomics to identify Vps35/retromer as a stimulus-dependent interacting partner of autophagosomes in human breast cancer cells [49]. A further test for autophagic response revealed that a significantly lower autophagic activity in the yeast *vps35* null mutant [49]. Another study identified that the mutant *VPS35* allele that causes Parkinson’s disease (PD), VPS35 D620N, also impairs autophagy and alters the trafficking of the multi-pass transmembrane autophagy protein Atg9A in mammalian cells [50]. However, none of these studies addressed the mechanism by which the retromer regulates autophagocytosis. Here, we provide genetic, live-cell imaging and biochemical evidence that MoVps35-GFP positively regulates the autophagy process via recycling or retrieving truncated MoAtg8 to the appropriate compartments, likely PAS, for regeneration of autophagosomes. In a previous report in *M*. *oryzae*, EGFP-MgAtg9 and DsRed2-MgAtg8 displayed significant colocalization [35], suggesting that these components interact in conidia. Although our data identified that MoAtg8 mislocalization is a likely contributor to the impaired autophagosome formation in the *ΔMovps35* mutant, it is conceivable that other MoAtg proteins are subjected to the retromer complex mediated retrograde transportation too. Given the conservation of the retromer and its role in autophagy, this mechanism likely provides a paradigm for a novel role of retromer in the regulation of autophagy in various eukaryotic organisms.

Autophagy plays an important role in recycling cellular components in eukaryotic cells. Although more than 30 genes have been described for involvement in autophagy, in yeast [13,14], the mechanisms for the activation of this process and for the recycling of its components for autophagosome formation remain poorly understood. A ubiquitin-like system mediates the conjugation of the cleaved Atg8 to the lipid phosphatidylethanolamine (PE), and this conjugate (Atg8PE) is then tethered to autophagosome membrane, where it is necessary for phagophore expansion during autophagosome formation [36,39]. In addition to the lipidation of Atg8, delipidation of Atg8 is also required for autophagosome maturation [41]. In yeast, the cleaved Atg8 has a C-terminal glycine residue that exists in two different forms: a lipidated (Atg8PE) and a deconjugated form [51]. The former is involved in autophagosome formation; the role of latter is to release outer membrane-bound Atg8 upon completion of the autophagosome, presumably for reuse in subsequent rounds of autophagosome formation [41]. However, how the deconjugated Atg8 gets to the PAS for autophagosome formation is currently unknown. Our data indicate a direct interaction between vacuolar membrane conjugated MoAtg8 and Vps35 and suggest a novel mechanism for lipidated MoAtg8 recycling via retromer-mediated transport in *M*. *oryzae*. It is possible that the retrieval machinery and/or the cargo-recognition complex of the retromer recognize the lipidated MoAtg8 as a specific cargo/substrate.

## Materials and Methods

### Fungal strains and growth conditions

All strains used in this study were listed in S2 Table. *M*. *oryzae* wild-type *Δku70* [15] and all mutant strains were grown on complete medium (CM), starch yeast medium (SYM), oatmeal agar medium (OAT) and rice-polish agar medium (RPA) for mycelial growth assays and on RPA medium for conidiation assays as previously described [52]. To test the sensitivity against cell-wall-disrupting agents, vegetative growth of fungal strains was monitored on CM plates with 200 μg/mL congo red (CR) or 200 μg/mL Calcofluor White (CFW) or 0.01% sodium dodecyl sulfate (SDS). For oxidative stress sensitivity assays, *M*. *oryzae* strains were grown on CM containing 5 mM H_2_O_2_ and the sensitivity was evaluated by measuring the colony diameter of 6-day-old cultures. Experimental results were verified with a minimum of two strains of the same genotype. All experiments were repeated at least three times.

### Generation of the gene replacement mutants

The *M*. *oryzae* protoplast preparation and fungal transformation were performed by following standard protocols [53]. Hygromycin- or neomycin-resistant transformants were selected on media supplemented with 250 μg/mL hygromycin B (Roche Applied Science) or 200 μg/mL G418 (Invitrogen).

To generate *ΔMovps35* mutant, a 1,176-bp fragment upstream from *MoVPS35* was amplified with primers MO05089AF and MO05089AR, and this amplicon was subsequently cloned into the *Xho*I and *Eco*RI sites upstream of the hph cassette on pCX63 [24]. Then, 1.14 kb fragment downstream of *MoVPS35* was amplified with primers MO05089BF and MO05089BR, and cloned into the *Bam*HI and *Xba*I sites downstream of *HPH* cassette, and this plasmid was transformed into protoplasts of the wild-type *Δku70* strain. Hygromycin-resistant transformants were screened by PCR with primers MO05089UA and H853 and primers MO05089OF and MO05089OR (S3 Table). At least two isolates that tested positive with PCR were further verified by Southern blot analysis performed with the DIG-High Prime DNA Labeling and Detection Starter Kit I Roche (Roche, Mannheim, Germany).

The split-marker approach was used to generate gene replacement constructs for other components of the retromer complex [54]. Primers used to amplify the flanking sequences of *MoVPS26* and *MoVPS29* are listed in S3 Table. Each construct was transformed into protoplasts of *Δku70* to generate the *ΔMovps26* and *ΔMovps29* deletion mutants (S2 Table). Putative knockout mutants were identified by PCR screening and confirmed by DNA gel blot analysis.

### Construction of GFP fusion vector and complementation

The MoVps35-GFP fusion vector, named pGM-MoVps35-GFP, was constructed by amplification of 5,027-bp fragment including 2,944-bp MoVps35 coding sequence and a 2,083-bp native promoter region using primers MO05089CF and MO05089CR (S3 Table). The 5,027-bp PCR product was then cloned into pGEM-T easy vector (Promega) to generate pGM-MoVps35. The GFP allele [55] was amplified using primers Bgl II-GFPF and Bgl II-GFPR (S3 Table), then cloned into pGEM-T easy vector. It was subsequently digested with *Bgl*II to release the GFP allele with *Bgl*II sticky ends, which was inserted into *Bgl*II site of pGM-MoVps35 to create pGM-MoVps35-GFP. We verified the orientation of GFP insertion and in-frame fusion by sequencing the pGM-MoVps35-GFP vector. To generate MoVps35-GFP strain, pGM-MoVps35-GFP construct was co-transformed into protoplasts of the target mutant along with a vector harboring neomycin-resistance marker (pKNT). Transformants carrying a single insertion were screened by PCR with requisite primer pairs (S3 Table) and further confirmed by Southern blot analysis. The same approach was used to generate gene fusion GFP constructs for other components of the retromer complex. Primers used to amplify the complementation sequences of *MoVPS26* and *MoVPS29* are listed in S3 Table.

### Appressorium formation and plant infection assays

For appressorial assays, conidia were harvested from 10-day-old OAT or RPA cultures. Aliquots (30 μL) of conidial suspensions (5×10^4^ conidia/mL in sterile water) were applied on the hydrophobic side of Gelbond film (Cambrex Bio Science) and incubated under humid conditions at room temperature. Conidial germination and appressorium formation were examined at 0.5, 1, 2, 4, 8 and 24 h post incubation. For penetration assays, conidial suspension in sterile water was inoculated on onion epidermal cells or barley leaf and assessed after 24 h, 48 h and 72 h. Penetration pegs and infection hyphae were detected by staining for papillary callose deposits using Aniline blue [56]. For pathogenicity assays, two-week-old seedlings of rice (*Oryza sativa* L.) cultivar CO39 were used for spray inoculation assays as described [52]. Eight-day-old seedlings of barley cultivar Golden Promise were also used for drop inoculation and mycelial plug assays on the non-wounded or wounded barley leaves [57].

### Cytological analysis

For glycogen staining, the *M*. *oryzae* conidia were inoculated on hydrophobic plastic coverslips for different time points and stained with a solution consisting of 60 mg/mL of KI and 10 mg/mL of I_2_ in distilled water [7]. Yellowish-brown glycogen deposits became visible immediately in bright field. For lipid bodies staining, samples were stained with Bodipy (D3922, Invitrogen) to detect neutral lipids. Bodipy was used at 10 μg/mL (stock 1 mg/mL in ethanol) in PBS buffer. All samples were examined and photographed by using an Olympus-BX51 fluorescence microscope with a cooled CCD camera (DP72, Olympus, Japan). Calcofluor White (Sigma-Aldrich, USA) was used at 3 μg/mL to visualize cell wall and septa of conidia. To visualize the vacuolar membrane, conidia, vegetative hyphae and germinated conidia were treated with 4 μg/mL FM4-64 solution for 30–60 min before observed under the confocal microscope. To examine the effects of microtubule inhibitor methyl 1-(butylcarbamoyl)-2-benzimidazolecarbamate (MBC) or the actin inhibitor latrunculin A (LatA) on trafficking ability of MoVps35 in cells, The MoVps35-GFP strain was inoculated on hydrophobic plastic coverslips and treated for 30 minutes with MBC (final concentration 10 μM) or LatA (final concentration 10 μM) at the hooking stage. A 0.1% DMSO solvent control was used in these assays.

### Molecular manipulations

Standard molecular manipulations were performed as described [58]. Total RNA was isolated from mycelia with TRIzol reagent (Invitrogen). Purified RNA was treated with DNase (Takara) and was verified as DNA free by using it directly as template in a PCR assay. First-strand cDNA was synthesized with the M-MLV reverse transcriptase (Invitrogen), and qRT-PCR was performed with the Eppendorf Mastercycler ep Realplex2 PCR system using SYBR Premix Ex TaqTM (RR420A, Takara). Primers used to amplify selected genes in qRT-PCR reactions are listed in S3 Table.

### Yeast two-hybrid assay

Yeast two-hybrid assay was carried out as indicated in MATCHMAKER GAL4 Two-Hybrid System 3 (Clontech). The full-length cDNA of *MoVPS35* was amplified with the primer pair MO05089BDF/MO05089BDR (S3 Table) and cloned into pGBKT7 as the bait vector BD-Movps35. The full-length cDNAs of *MoVPS26* and *MoVPS29* were amplified with the primer pairs MO04830ADF/MO04830ADR and MO02524ADF/MO02524ADR (S3 Table), respectively, and were cloned into pGADT7 as the prey vectors AD-Movps26 and AD-Movps29. The resultant bait and prey vectors were confirmed by sequencing and were co-transformed into the yeast strain AH109. The Leu^+^ and Trp^+^ yeast transformants were isolated and assayed for growth on SD-Trp-Leu-His-Ade medium at specified concentrations. Yeast strains for positive and negative controls were as described in the Matchmaker kit.

### Immunoblot and immunoprecipitation analysis

For total protein extraction, mycelia grown in liquid CM and MM-N medium (used for nitrogen starvation, 0.5 g/L KCl, 0.5 g/L MgSO_4_, 1.5 g/L KH_2_PO4, 10 g/L glucose, pH 6.5) were ground into a fine powder in liquid nitrogen and resuspended in 0.6 mL extraction buffer (50 mM Tris-HCl (pH 7.4), 150 mM NaCl, 1 mM EDTA, 2 mM PMSF and 1% Triton X-100). The supernatants were centrifuged 15,000 g for 25 min at 4°C to remove cell debris. Total protein concentration was measured using the Bio-Rad Protein Assay and separated on a 12.5% SDS PAGE gel and transferred to PVDF membranes for Western blot analysis. The expression of RFP-Atg8 was detected with anti-RFP (Clontech, USA). The horseradish peroxidase–conjugated secondary antibody and the ECL kit (Amersham Biosciences, Germany) were used to detect the chemiluminescent signals.

For the immunoprecipitation of GFP-fusion-proteins from cellular extracts, equal concentration of total proteins were isolated and incubated with 20–30 μL of GFP-Trap agarose beads (ChromoTek, Germany) and carried out as manufacturer’s instructions. Proteins eluted from GFP-Trap agarose beads were analyzed by immunoblot detection with the anti-RFP (Clontech, USA), anti-GFP (Sigma-Aldrich) antibodies and anti-Actin (Sigma-Aldrich).

### Live cell imaging of *M*. *oryzae*

Conventional epifluorescence and differential interference contrast (DIC) microscopy was performed with a Olympus BX51 microscope (Olympus, Japan), using a 40x 1.3 NA (numerical aperture), 60x 1.35 NA or 100x 1.40 NA Olympus oil immersion objective lens. Images were acquired using an Olympus DP72 camera and analyzed with DT2-BSW image-processing software. Fluorescence was observed with Olympus U-RFL-T mercury lamp source. The filter sets used were: DAPI, GFP and RFP or FM4-64. Alternatively, confocal microscopy was used for time-lapse or live cell fluorescence imaging by using the Nikon TiE system (Nikon, Japan) as described [59]. The elapsed time is indicated in seconds. Image processing and figure preparation was performed using Image J, Adobe Photoshop, PowerPoint and Microsoft Excel.

### Accession numbers

Sequence data from this article can be found in the GenBank/EMBL databases under the following accession numbers: *S*. *cerevisiae VPS35* (NP_012381), *S*. *cerevisiae VPS29* (NP_011876), *S*. *cerevisiae VPS26* (NP_012482), *S*. *cerevisiae VPS17* (NP_014775), *S*. *cerevisiae VPS5* (NP_014712), *M*. *oryzae MoVPS35* (XP_003712611), *M*. *oryzae MoVPS29* (XP_003709334), *M*. *oryzae MoVPS26* (XP_003713759), *M*. *oryzae MoVPS17* (XP_003714383), *M*. *oryzae MoVPS5* (XP_003709457).

## Supporting Information

S1 Fig Generation and characterization of *ΔMovps35* deletion mutant. (A) Schematic diagram of the genomic region of the *MoVPS35* and *HPH* genes. Primers F1 (MO05089AF), R1 (MO05089AR), F2 (MO05089BF) and R2 (MO05089BR) were used to generate *MoVPS35* gene replacement constructs, and OF1 (MO05089OF), OR1 (MO05089OR), F1 (MO05089AF) and R1 (MO05089AR) were used for mutant screening and identification. H, *Hin*d III. (B) DNA gel blots of *Hin*d III-digested genomic DNA were hybridized with *MoVPS35* upstream fragment as the probe. A 2.12-kb band was observed in the wild type, while 3.494-kb bands were observed in the two independent mutants and complementation strain. *ΔKu70*, wild-type strain, *ΔMovps35-42* and *ΔMovps35-75*, null mutant, *ΔMovps35-Com*, complementation strain. (C) The *ΔMovps35* mutant displayed reduced mycelial growth on CM, SYM, OAM and RPA medium. (D) Development of conidia on conidiophores was significantly reduced in the *ΔMovps35* mutant. Bar = 50 μm. (E) Analysis of conidia production. The respective strains were initially grown in the dark for a day followed by exposure to constant illumination for 14 day on RPA plate (diameter 9 cm). Data represents mean ± SD based on three independent replicates, and double asterisks indicate statistically significant differences (P < 0.01). (F) Analysis of conidiophore formation. The number of conidiophores produced by the indicated strain per microscopic field was quantified at 24 h post photo induction. Results were quantified in three independent replicates and represented as mean ± SD, and double asterisks indicate statistically significant differences (P < 0.01).
(TIF)

S2 Fig *ΔMovps35* mutant is not sensitive to H_2_O_2_ but is involved in the tolerance to cell wall or membrane stress agents. (A) The wild type, *ΔMovps35* and the complemented strain were incubated on CM medium supplemented with various stress inducers for 6 days at 28 ^0^C. (B) Analysis of the growth inhibition rate in mycelia in CM supplemented with various stress inducers. Data comprise three independent experiments with triple replications in each instance.
(TIF)

S3 Fig The *MoVPS26* and *MoVPS29* gene-replacement construct and mutant. (A) Schematic diagram of the genomic region of the *MoVPS26* and *HPH* genes. Primers F1 (MO04830AF), R1 (MO04830AR), F2 (MO04830BF) and R2 (MO04830BR) were used to generate *MoVPS35* gene replacement constructs, and OF1 (MO04830OF), OR1 (MO04830OR), F1 (MO04830AF) and R1 (MO04830AR) were used for mutant screening and identification. E, *Eco*RV. (B) DNA gel blots of *Eco*RV -digested genomic DNA were hybridized with *MoVPS26* upstream fragment as the probe. A 1.797-kb band was observed in the wild type and complementation strain, while 8.993-kb bands were observed in the two independent mutants and complementation strain. *ΔKu70*, wild-type strain, *ΔMovps26-9* and *ΔMovps26-8*, null mutant, *ΔMovps26-Com*, complementation strain. (C) Schematic diagram of the genomic region of the *MoVPS29* and *HPH* genes. Primers F1 (MO02524AF), R1 (MO02524AR), F2 (MO02524BF) and R2 (MO02524BR) were used to generate *MoVPS29* gene replacement constructs, and OF1 (MO02524OF), OR1 (MO02524OR), F1 (MO02524AF) and R1 (MO02524AR) were used for mutant screening and identification. N, *Nco* I. (D) DNA gel blots of *Nco* I-digested genomic DNA were hybridized with *MoVPS29* upstream fragment as the probe. A 1.566-kb band was observed in the wild type and complementation strain, while 1.966-kb bands were observed in the two independent mutants and complementation strain. *ΔKu70*, wild-type strain, *ΔMovps29-2* and *ΔMovps29-23*, null mutant, *ΔMovps29-Com*, complementation strain.
(TIF)

S4 Fig MoVps26 and MoVps29 are required for conidiation in *M*. *oryzae*. (A) Development of conidia on conidiophores was significantly reduced in *ΔMovps26* and *ΔMovps29* mutants. Scale bar = 50 μm. (B) Analysis of conidia production. The respective strains were initially grown in the dark for a day followed by exposure to constant illumination for 14 day on RPA plate (diameter 9 cm). Data represents mean ± SD based on three independent replicates, and double asterisks indicate statistically significant differences (P < 0.01).
(TIF)

S5 Fig The *ΔMovps26* and *ΔMovps29* are impaired in glycogen distribution, lipid droplet turnover and appressorial turgor generation. (A) Conidia from wild type (WT), *ΔMovps26* or *ΔMovps26* strain were germinated on hydrophobic gelbond membranes. Drop of water was replaced by iodine solution at 0 h, 8 h, 16 h and 24 h to stain for and microscopically visualize glycogen as yellowish-brown deposits. (B) Quantitation of the total glycogen content in conidia during pathogenic development in the indicated strains. (C) MoVps26 and MoVps29 are involved in lipid body translocation and degradation during appressorium morphogenesis. Conidia of *M*. *oryzae* WT, *ΔMovps26* and *ΔMovps29* were incubated in water droplets on the hydrophobic surface of gelbond and allowed to form appressoria for up to 24 h. Samples were removed at 0, 8, 16 and 24 h and stained with Bodipy to visualize lipid bodies by confocal microscopy. (D) Quantitative analysis of lipid bodies during appressorium morphogenesis. The bar charts show the mean and standard deviation from three independent replicates of the experiment. (E) Appressorium turgor was measured by incipient cytorrhysis assays. Appressoria were allowed to form on plastic coverslips for 24 h, and the collapsed appressoria assessed after exposure to 2 M glycerol solutions. White arrows indicate the appressoria in the WT, *ΔMovps26* or *ΔMovps26* strain. (F) Proportion of collapsed appressoria after exposure of conidia to 2 M glycerol. Bars = 20 μm.
(TIF)

S6 Fig Subcellular localization of autophagosomes during infection-related development. (A) Conidia from RFP-MoAtg8 expressed in the *ΔMoatg8* or *ΔMovps35* strain were inoculated onto glass coverslips and observed by epifluorescence microscopy at the indicated times. RFP-MoAtg8 punctate structures (arrows and arrowheads) were significantly reduced in number in both germ tubes and appressoria of *ΔMovps35 RFP-MoATG8* when compared to *ΔMoatg8 RFP-MoATG8*. Bar = 10 μm. (B) Bar chart showing ratio of punctate autophagosomes present in germ tubes and appressoria 2 h, 4 h, 8 h and 24 h after inoculation. Values represent mean and standard deviation from three independent replicates using 200 conidia per sample. Double asterisks indicate statistically significant differences (P < 0.01).
(TIF)

S7 Fig Localization of MoVps35-GFP during different stages of appressorium morphogenesis. Conidia were allowed to form appressoria and localization of MoVps35-GFP observed during 48 h using an Olympus BX-51 epifluorescence microscope. Fusion proteins localized to cytoplasm as small punctae in the conidium, germ tube and appressorium. BF = Bright field. Scale bar = 10 μm.
(TIF)

S8 Fig Mobility of MoVps35-GFP is dependent on microtubules not F-actin microfilaments. Actin polymerization inhibitor LatA and microtubule cytoskeleton inhibitor MBC were added to developing appressoria, respectively. Fluorescently labeled motile punctate compartments lost the ability to carry out long distance transport in fungal cells treated with MBC. The mobility of MoVps35-GFP was not visibly impaired after treatment with LatA. DMSO-treated sample served as a control. Arrowheads indicate the relative position of punctate compartments at each time point. Elapsed time indicated in seconds. See also S4–S6 Movies. Scale bars = 10 μm.
(TIF)

S9 Fig Localization of MoVps26-GFP and MoVps29-GFP during different stages of appressorium morphogenesis. Conidia were allowed to form appressoria and localization of MoVps26-GFP or MoVps29-GFP observed during 48 h using an Olympus BX-51 epifluorescence microscope. Fusion proteins were apparent as small cytosolic punctae in conidia, germ tubes and appressoria. BF = Bright field. Scale bar = 10 μm.
(TIF)

S10 Fig Close associations between MoVps35-GFP and RFP-MoAtg8 during different stages of development in *M*. *oryzae*. Live cell imaging of distribution and dynamics of MoVps35-GFP and RFP-MoAtg8 pathogenic and vegetative growth in *M*. *oryzae*. The dotted box (left panel) and enlarged dotted box (right panel) highlight compartments showing co-localized MoVps35-GFP and RFP-MoAtg8. White arrow in the inset shows the path for fluorescence intensity distribution by line-scan analysis. Images on the left panel are merged MoVps35-GFP (green) and RFP-MoAtg8 (red), MoVps35-GFP (green) alone, RFP-MoAtg8 (red) alone, and DIC. Bar = 10 μm. (A) conidial germination stage. (B) appressorium initiation. (C) mycelial growth under nitrogen stravation condition.
(TIF)

S11 Fig Assays for the expression of *MoATG8* and *MoATG4* genes in the *ΔMovps35* mutant and phenotypic characterization of *ΔMovps35-ATG8OE* strains. (A) The expression levels of *MoATG8* and *MoATG4* genes were assayed by qRT-PCR with RNA isolated from vegetative hyphae culturing on MM-N medium. (B) qRT-PCR-based confirmation of *MoATG8* transcript levels in *ΔMovps35-ATG8OE* strains. (C) Morphology of the colonies of wild type, *ΔMovps35* and *ΔMovps35-ATG8OE* strain. Analysis of conidiation (D), diameter of the colonies (E) and pathogenicity (F) in wild type, *ΔMovps35* and *ΔMovps35-ATG8OE* strain.
(TIF)

S1 Table Putative retromer components in *M*. *oryzae* identified by BLASTP analysis. (DOCX)

S2 Table Wild-type and mutant strains of fungi used in this study. (DOC)

S3 Table Oligonucleotide primers used in this study. (DOC)

S1 Movie Cellular dynamics of autophagosomes during infection-related development in an *ΔMoatg8 RFP-MoATG8* strain. Movie was taken by Nikon TiE system (CFI Plan Apochromat VC 100XH 1.4 N.A. objective) equipped with a Yokogawa CSU-X1-A1 spinning disk unit, elapsed time is indicated in seconds.
(MOV)

S2 Movie Cellular dynamics of autophagosomes during infection-related development in *ΔMovps35 RFP-MoATG8*. Movie was taken by Nikon TiE system (CFI Plan Apochromat VC 100XH 1.4 N.A. objective) equipped with a Yokogawa CSU-X1-A1 spinning disk unit, elapsed time is indicated in seconds.
(MOV)

S3 Movie Dynamics and mobility of MoVps35-GFP. Small punctae in germinating conidia of MoVps35-GFP move fast in cells, and always associate with the vacuolar membrane. Movie was taken with a Nikon TiE system (CFI Plan Apochromat VC 100XH 1.4 N.A. objective) equipped with a Yokogawa CSU-X1-A1 spinning disk unit. Elapsed time is indicated in seconds.
(MOV)

S4 Movie Mobility of MoVps35-GFP after treat with MBC. Movie was taken using a Nikon TiE system (CFI Plan Apochromat VC 100XH 1.4 N.A. objective) equipped with a Yokogawa CSU-X1-A1 spinning disk unit. Elapsed time is indicated in seconds.
(MOV)

S5 Movie Mobility of MoVps35-GFP after treat with LatA. Movie was taken using a Nikon TiE system (CFI Plan Apochromat VC 100XH 1.4 N.A. objective) equipped with a Yokogawa CSU-X1-A1 spinning disk unit. Elapsed time is indicated in seconds.
(MOV)

S6 Movie Mobility of MoVps35-GFP after treat with DMSO. Movie was taken using Nikon TiE system (CFI Plan Apochromat VC 100XH 1.4 N.A. objective) equipped with a Yokogawa CSU-X1-A1 spinning disk unit. Elapsed time is indicated in seconds.
(MOV)

S7 Movie Dynamics and mobility of MoVps26-GFP. Small punctae in germinal conidium of MoVps26-GFP move very fast in the cells, and always associate with the membrane of vacuole. Movie was taken by Nikon TiE system (CFI Plan Apochromat VC 100XH 1.4 N.A. objective) equipped with a Yokogawa CSU-X1-A1 spinning disk unit. Elapsed time is indicated in seconds.
(MOV)

S8 Movie Dynamics and mobility of MoVps29-GFP. Small punctae in germinal conidium of MoVps29-GFP move very fast in the cells, and always associate with the membrane of vacuole. Movie was taken by Nikon TiE system (CFI Plan Apochromat VC 100XH 1.4 N.A. objective) equipped with a Yokogawa CSU-X1-A1 spinning disk unit. Elapsed time is indicated in seconds.
(MOV)

S9 Movie A three-dimensional image reconstruction of dual-labeled *ΔMoatg8 RFP-MoATG8 MoVps35-GFP* strain is shown in a rotational view. Punctate MoVps35-GFP colocalized with the RFP-MoAtg8 at several interfaces. Serial sections of a conidia were recorded using a Nikon A1 confocal microscope.
(MOV)

S10 Movie Close association of MoVps35-GFP labeled vesicles and RFP-MoAtg8 compartments. Association of a mobile MoVps35-GFP labeled vesicle occurs with an autophagosome labeled with RFP-MoAtg8 (arrowheads) in a conidium. Movement of a RFP-MoAtg8 labeled vesicle with apparent dissociation (the first arrowhead marked). Other instances of MoVps35-GFP labeled vesicles interacting with RFP-MoAtg8 labeled autophagosome were also observed (the second arrowhead marked). Movie was taken by Nikon A1 confocal microscope. Elapsed time is indicated in millisecond.
(MOV)

## References

[1] Ebbole DJ (2007) Magnaporthe as a model for understanding host-pathogen interactions. Annual Review of Phytopathology 45 437–456. 17489691 10.1146/annurev.phyto.45.062806.094346

[2] Talbot NJ (2003) On the trail of a cereal killer: Exploring the biology of Magnaporthe grisea. Annual Review of Microbiology 57: 177–202. 14527276 10.1146/annurev.micro.57.030502.090957

[3] Bourett TM, Howard RJ (1991) In vitro development of penetration structures in the rice blast fungus Magnaporthe grisea. Can J Bot 68: 329–342.

[4] Caracuel-Rios Z, Talbot NJ (2007) Cellular differentiation and host invasion by the rice blast fungus Magnaporthe grisea. Current opinion in microbiology 10: 339–345. 17707684 10.1016/j.mib.2007.05.019

[5] Veneault-Fourrey C, Barooah M, Egan M, Wakley G, Talbot NJ (2006) Autophagic fungal cell death is necessary for infection by the rice blast fungus. Science 312: 580–583. 16645096 10.1126/science.1124550

[6] Veneault-Fourrey C, Talbot NJ (2007) Autophagic cell death and its importance for fungal developmental biology and pathogenesis. Autophagy 3: 126–127. 17172805 10.4161/auto.3529

[7] Thines E, Weber RW, Talbot NJ (2000) MAP kinase and protein kinase A-dependent mobilization of triacylglycerol and glycogen during appressorium turgor generation by Magnaporthe grisea. The Plant cell 12: 1703–1718. 11006342 10.1105/tpc.12.9.1703 PMC149080

[8] Wang ZY, Jenkinson JM, Holcombe LJ, Soanes DM, Veneault-Fourrey C, et al (2005) The molecular biology of appressorium turgor generation by the rice blast fungus Magnaporthe gasea. Biochemical Society Transactions 33: 384–388. 15787612 10.1042/BST0330384

[9] de Jong JC, McCormack BJ, Smirnoff N, Talbot NJ (1997) Glycerol generates turgor in rice blast. Nature 389: 244–244.

[10] Kankanala P, Czymmek K, Valent B (2007) Roles for rice membrane dynamics and plasmodesmata during biotrophic invasion by the blast fungus. Plant Cell 19: 706–724. 17322409 10.1105/tpc.106.046300 PMC1867340

[11] Pollack JK, Harris SD, Marten MR (2009) Autophagy in filamentous fungi. Fungal Genetics and Biology 46: 1–8. 10.1016/j.fgb.2008.10.010 19010432

[12] Voigt O, Poeggeler S (2013) Self-eating to grow and kill: autophagy in filamentous ascomycetes. Applied Microbiology and Biotechnology 97: 9277–9290. 24077722 10.1007/s00253-013-5221-2

[13] Klionsky DJ, Cregg JM, Dunn WA, Emr SD, Sakai Y, et al (2003) A unified nomenclature for yeast autophagy-related genes. Developmental Cell 5: 539–545. 14536056 10.1016/s1534-5807(03)00296-x

[14] Ohsumi Y (2001) Molecular dissection of autophagy: two ubiquitin-like systems. Nature reviews Molecular cell biology 2: 211–216. 11265251 10.1038/35056522

[15] Kershaw MJ, Talbot NJ (2009) Genome-wide functional analysis reveals that infection-associated fungal autophagy is necessary for rice blast disease. Proceedings of the National Academy of Sciences of the United States of America 106: 15967–15972. 10.1073/pnas.0901477106 19717456 PMC2747227

[16] Deng YZ, Ramos-Pamplona M, Naqvi NI (2009) Autophagy-assisted glycogen catabolism regulates asexual differentiation in Magnaporthe oryzae. Autophagy 5: 33–43. 19115483 10.4161/auto.5.1.7175

[17] Koumandou VL, Klute MJ, Herman EK, Nunez-Miguel R, Dacks JB, et al (2011) Evolutionary reconstruction of the retromer complex and its function in Trypanosoma brucei. Journal of Cell Science 124: 1496–1509. 10.1242/jcs.081596 21502137 PMC3078816

[18] Seaman MN, McCaffery JM, Emr SD (1998) A membrane coat complex essential for endosome-to-Golgi retrograde transport in yeast. The Journal of cell biology 142: 665–681. 9700157 10.1083/jcb.142.3.665 PMC2148169

[19] Burd C, Cullen PJ (2014) Retromer: A Master Conductor of Endosome Sorting. Cold Spring Harbor Perspectives in Biology 6. 10.1101/cshperspect.a016774 PMC3941235 24492709

[20] Seaman MNJ (2012) The retromer complex—endosomal protein recycling and beyond. Journal of Cell Science 125: 4693–4702. 10.1242/jcs.103440 23148298 PMC3517092

[21] Maruzs T, Lorincz P, Szatmari Z, Szeplaki S, Sandor Z, et al (2015) Retromer Ensures the Degradation of Autophagic Cargo by Maintaining Lysosome Function in Drosophila. Traffic. 10.1111/tra.12309 26172538

[22] Jeon J, Goh J, Yoo S, Chi M-H, Choi J, et al (2008) A putative MAP kinase kinase kinase, MCK1, is required for cell wall integrity and pathogenicity of the rice blast fungus, Magnaporthe oryzae. Molecular Plant-Microbe Interactions 21: 525–534. 10.1094/MPMI-21-5-0525 18393612

[23] Liu W, Xie S, Zhao X, Chen X, Zheng W, et al (2010) A Homeobox Gene Is Essential for Conidiogenesis of the Rice Blast Fungus Magnaporthe oryzae. Molecular Plant-Microbe Interactions 23: 366–375. 10.1094/MPMI-23-4-0366 20192824

[24] Zheng W, Zhao X, Xie Q, Huang Q, Zhang C, et al (2012) A Conserved Homeobox Transcription Factor Htf1 Is Required for Phialide Development and Conidiogenesis in Fusarium Species. Plos One 7. 10.1371/journal.pone.0045432 PMC3448628 23029006

[25] Seaman MNJ (2004) Cargo-selective endosomal sorting for retrieval to the Golgi requires retromer. Journal of Cell Biology 165: 111–122. 15078902 10.1083/jcb.200312034 PMC2172078

[26] Seaman MNJ (2005) Recycle your receptors with retromer. Trends in Cell Biology 15: 68–75. 15695093 10.1016/j.tcb.2004.12.004

[27] Howard RJ, Ferrari MA, Roach DH, Money NP (1991) Penetration of hard substrates by a fungus employing enormous turgor pressures. Proceedings of the National Academy of Sciences of the United States of America 88: 11281–11284. 1837147 10.1073/pnas.88.24.11281 PMC53118

[28] Badaruddin M, Holcombe LJ, Wilson RA, Wang Z-Y, Kershaw MJ, et al (2013) Glycogen Metabolic Genes Are Involved in Trehalose-6-Phosphate Synthase-Mediated Regulation of Pathogenicity by the Rice Blast Fungus Magnaporthe oryzae. Plos Pathogens 9. 10.1371/journal.ppat.1003604 PMC3789717 24098112

[29] Liu X-H, Lu J-P, Zhang L, Dong B, Min H, et al (2007) Involvement of a Magnaporthe grisea serine/threonine kinase gene, MgATG1, in appressorium turgor and pathogenesis. Eukaryotic Cell 6: 997–1005. 17416896 10.1128/EC.00011-07 PMC1951528

[30] Deng YZ, Naqvi NI (2010) A vacuolar glucoamylase, Sga1, participates in glycogen autophagy for proper asexual differentiation in Magnaporthe oryzae. Autophagy 6: 455–461. 10.4161/auto.6.4.11736 20383057

[31] Liu T-B, Liu X-H, Lu J-P, Zhang L, Min H, et al (2010) The cysteine protease MoAtg4 interacts with MoAtg8 and is required for differentiation and pathogenesis in Magnaporthe oryzae. Autophagy 6: 74–85. 19923912 10.4161/auto.6.1.10438

[32] Liu X-H, Lu J-P, Lin F-C (2007) Autophagy during conidiation, conidial germination and turgor generation in Magnaporthe grisea. Autophagy 3: 472–473. 17495517 10.4161/auto.4339

[33] Lu J-P, Liu X-H, Feng X-X, Min H, Lin F-C (2009) An autophagy gene, MgATG5, is required for cell differentiation and pathogenesis in Magnaporthe oryzae. Current Genetics 55: 461–473. 10.1007/s00294-009-0259-5 19629489

[34] Deng Y, Qu Z, Naqvi NI (2012) Role of macroautophagy in nutrient homeostasis during fungal development and pathogenesis. Cells 1: 449–463. 10.3390/cells1030449 24710485 PMC3901100

[35] Dong B, Liu X-H, Lu J-P, Zhang F-S, Gao H-M, et al (2009) MgAtg9 trafficking in Magnaporthe oryzae. Autophagy 5: 946–953. 19556868 10.4161/auto.5.7.9161

[36] Kraft C, Martens S (2012) Mechanisms and regulation of autophagosome formation. Current Opinion in Cell Biology 24: 496–501. 10.1016/j.ceb.2012.05.001 22664348

[37] Rubinsztein DC, Shpilka T, Elazar Z (2012) Mechanisms of Autophagosome Biogenesis. Current Biology 22: R29–R34. 10.1016/j.cub.2011.11.034 22240478

[38] Klionsky DJ, Abdalla FC, Abeliovich H, Abraham RT, Acevedo-Arozena A, et al (2012) Guidelines for the use and interpretation of assays for monitoring autophagy. Autophagy 8: 445–544. 22966490 10.4161/auto.19496 PMC3404883

[39] Klionsky DJ, Abeliovich H, Agostinis P, Agrawal DK, Aliev G, et al (2008) Guidelines for the use and interpretation of assays for monitoring autophagy in higher eukaryotes. Autophagy 4: 151–175. 18188003 10.4161/auto.5338 PMC2654259

[40] Mizushima N (2006) Methods for monitoring macroautophagy. Tanpakushitsu kakusan koso Protein, nucleic acid, enzyme 51: 1542–1548. 16922435

[41] Nair U, Yen WL, Mari M, Cao Y, Xie Z, et al (2012) A role for Atg8-PE deconjugation in autophagosome biogenesis. Autophagy 8: 780–793. 10.4161/auto.19385 22622160 PMC3378420

[42] Pinar M, Pantazopoulou A, Penalva MA (2013) Live-cell imaging of Aspergillus nidulans autophagy RAB1 dependence, Golgi independence and ER involvement. Autophagy 9: 1024–1043. 10.4161/auto.24483 23722157 PMC3722313

[43] Nodzynski T, Feraru MI, Hirsch S, De Rycke R, Niculaes C, et al (2013) Retromer Subunits VPS35A and VPS29 Mediate Prevacuolar Compartment (PVC) Function in Arabidopsis. Molecular Plant 6: 1849–1862. 10.1093/mp/sst044 23770835

[44] Oliviusson P, Heinzerling O, Hillmer S, Hinz G, Tse YC, et al (2006) Plant retromer, localized to the prevacuolar compartment and microvesicles in Arabidopsis, may interact with vacuolar sorting receptors. Plant Cell 18: 1239–1252. 16582012 10.1105/tpc.105.035907 PMC1456867

[45] Yamazaki M, Shimada T, Takahashi H, Tamura K, Kondo M, et al (2008) Arabidopsis VPS35, a retromer component, is required for vacuolar protein sorting and involved in plant growth and leaf senescence. Plant and Cell Physiology 49: 142–156. 10.1093/pcp/pcn006 18222962

[46] Jaillais Y, Santambrogio M, Rozier F, Fobis-Loisy I, Miege C, et al (2007) The retromer protein VPS29 links cell polarity and organ initiation in plants. Cell 130: 1057–1070. 17889650 10.1016/j.cell.2007.08.040

[47] Korolchuk VI, Schutz MM, Gomez-Llorente C, Rocha J, Lansu NR, et al (2007) Drosophila Vps35 function is necessary for normal endocytic trafficking and actin cytoskeleton organisation. J Cell Sci 120: 4367–4376. 18057029 10.1242/jcs.012336

[48] Groppelli E, Len AC, Granger LA, Jolly C (2014) Retromer regulates HIV-1 envelope glycoprotein trafficking and incorporation into virions. PLoS Pathog 10: e1004518 10.1371/journal.ppat.1004518 25393110 PMC4231165

[49] Dengjel J, Hoyer-Hansen M, Nielsen MO, Eisenberg T, Harder LM, et al (2012) Identification of Autophagosome-associated Proteins and Regulators by Quantitative Proteomic Analysis and Genetic Screens. Molecular & Cellular Proteomics 11. 10.1074/mcp.M111.014035 PMC3316729 22311637

[50] Zavodszky E, Seaman MNJ, Moreau K, Jimenez-Sanchez M, Breusegem SY, et al (2014) Mutation in VPS35 associated with Parkinson's disease impairs WASH complex association and inhibits autophagy. Nature Communications 5. 10.1038/ncomms4828 PMC4024763 24819384

[51] Yu ZQ, Ni T, Hong B, Wang HY, Jiang FJ, et al (2012) Dual roles of Atg8-PE deconjugation by Atg4 in autophagy. Autophagy 8: 883–892. 10.4161/auto.19652 22652539 PMC3427254

[52] Chen J, Zheng W, Zheng S, Zhang D, Sang W, et al (2008) Rac1 Is Required for Pathogenicity and Chm1-Dependent Conidiogenesis in Rice Fungal Pathogen Magnaporthe grisea. Plos Pathogens 4. 10.1371/journal.ppat.1000202 PMC2575402 19008945

[53] Sweigard JA, Carroll AM, Farrall L, Chumley FG, Valent B (1998) Magnaporthe grisea pathogenicity genes obtained through insertional mutagenesis. Molecular plant-microbe interactions: MPMI 11: 404–412. 9574508 10.1094/MPMI.1998.11.5.404

[54] Catlett NL, Lee B-N, Yoder OC, Turgeon BG (2003) Split-marker recombination for efficient targeted deletion of fungal genes. Fungal Genet Newsl 50: 9–11.

[55] Chiu W, Niwa Y, Zeng W, Hirano T, Kobayashi H, et al (1996) Engineered GFP as a vital reporter in plants. Current biology: CB 6: 325–330. 8805250 10.1016/s0960-9822(02)00483-9

[56] Vogel J, Somerville S (2000) Isolation and characterization of powdery mildew-resistant Arabidopsis mutants. Proceedings of the National Academy of Sciences of the United States of America 97: 1897–1902. 10677553 10.1073/pnas.030531997 PMC26533

[57] Park G, Bruno KS, Staiger CJ, Talbot NJ, Xu JR (2004) Independent genetic mechanisms mediate turgor generation and penetration peg formation during plant infection in the rice blast fungus. Molecular Microbiology 53: 1695–1707. 15341648 10.1111/j.1365-2958.2004.04220.x

[58] Sambrook J, Fritsch E, Maniatis T (1989) Molecular Cloning:A laboratory Manual. Cold Spring Harbor, NY: Cold Spring Laboratory Press.

[59] Ramanujam R, Calvert ME, Selvaraj P, Naqvi NI (2013) The Late Endosomal HOPS Complex Anchors Active G-Protein Signaling Essential for Pathogenesis in Magnaporthe oryzae. Plos Pathogens 9. 10.1371/journal.ppat.1003527 PMC3731250 23935502

