# Supplementary figures and images for: Retromer Is Essential for Autophagy-Dependent Plant Infection by the Rice Blast Fungus
Source: PLoS Genet. 2015 Dec 10;11(12):e1005704. doi: 10.1371/journal.pgen.1005704 (PMC4686016; doi:10.1371/journal.pgen.1005704)

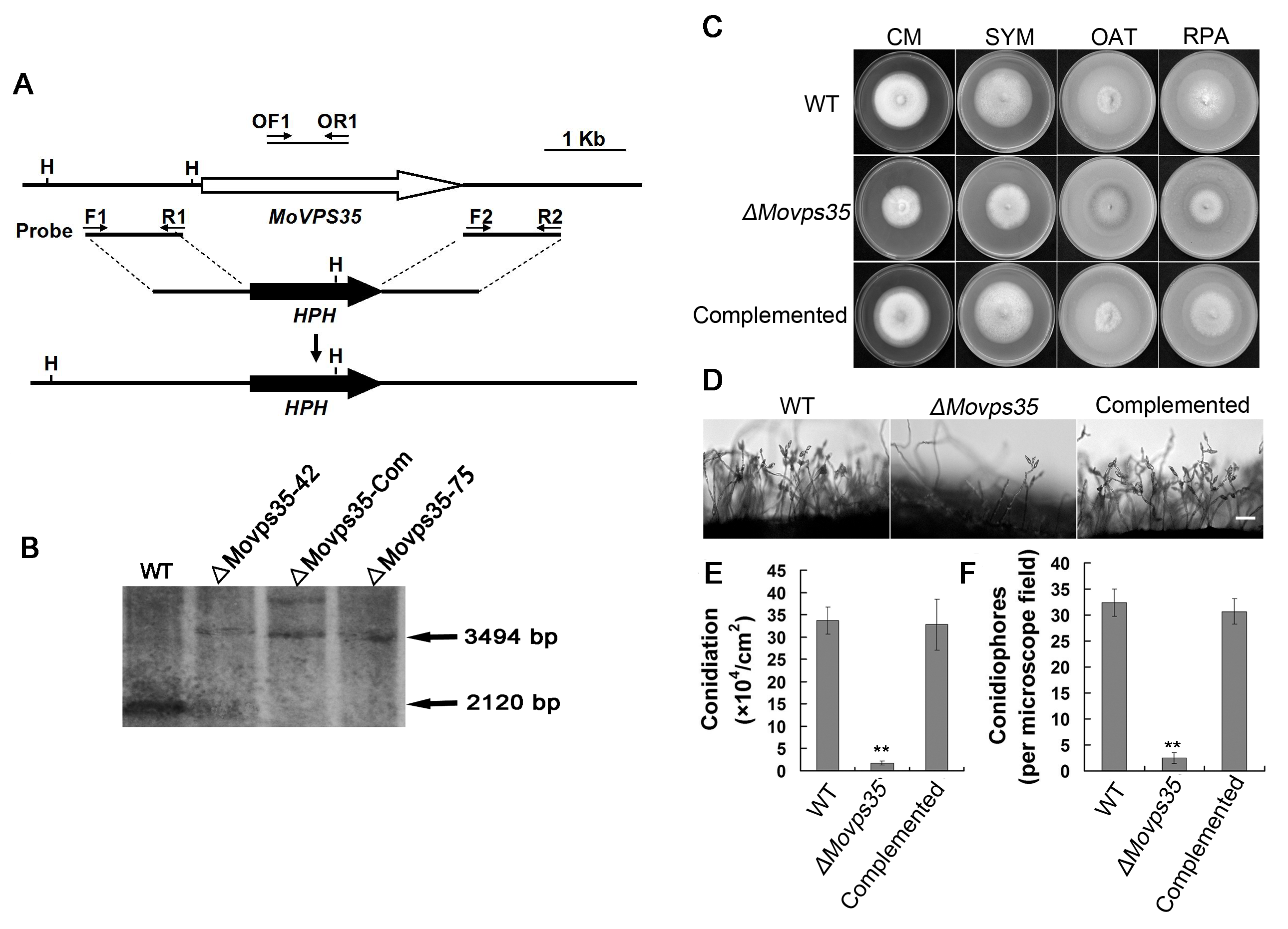

Supplement: S1 Fig — (A) Schematic diagram of the genomic region of the MoVPS35 and HPH genes. Primers F1 (MO05089AF), R1 (MO05089AR), F2 (MO05089BF) and R2 (MO05089BR) were used to generate MoVPS35 gene replacement constructs, and OF1 (MO05089OF), OR1 (MO05089OR), F1 (MO05089AF) and R1 (MO05089AR) were used for mutant screening and identification. H, Hind III. (B) DNA gel blots of Hind III-digested genomic DNA were hybridized with MoVPS35 upstream fragment as the probe. A 2.12-kb band was observed in the wild type, while 3.494-kb bands were observed in the two independent mutants and complementation strain. ΔKu70, wild-type strain, ΔMovps35-42 and ΔMovps35-75, null mutant, ΔMovps35-Com, complementation strain. (C) The ΔMovps35 mutant displayed reduced mycelial growth on CM, SYM, OAM and RPA medium. (D) Development of conidia on conidiophores was significantly reduced in the ΔMovps35 mutant. Bar = 50 μm. (E) Analysis of conidia production. The respective strains were initially grown in the dark for a day followed by exposure to constant illumination for 14 day on RPA plate (diameter 9 cm). Data represents mean ± SD based on three independent replicates, and double asterisks indicate statistically significant differences (P < 0.01). (F) Analysis of conidiophore formation. The number of conidiophores produced by the indicated strain per microscopic field was quantified at 24 h post photo induction. Results were quantified in three independent replicates and represented as mean ± SD, and double asterisks indicate statistically significant differences (P < 0.01). (TIF) [file pgen.1005704.s001.tif]

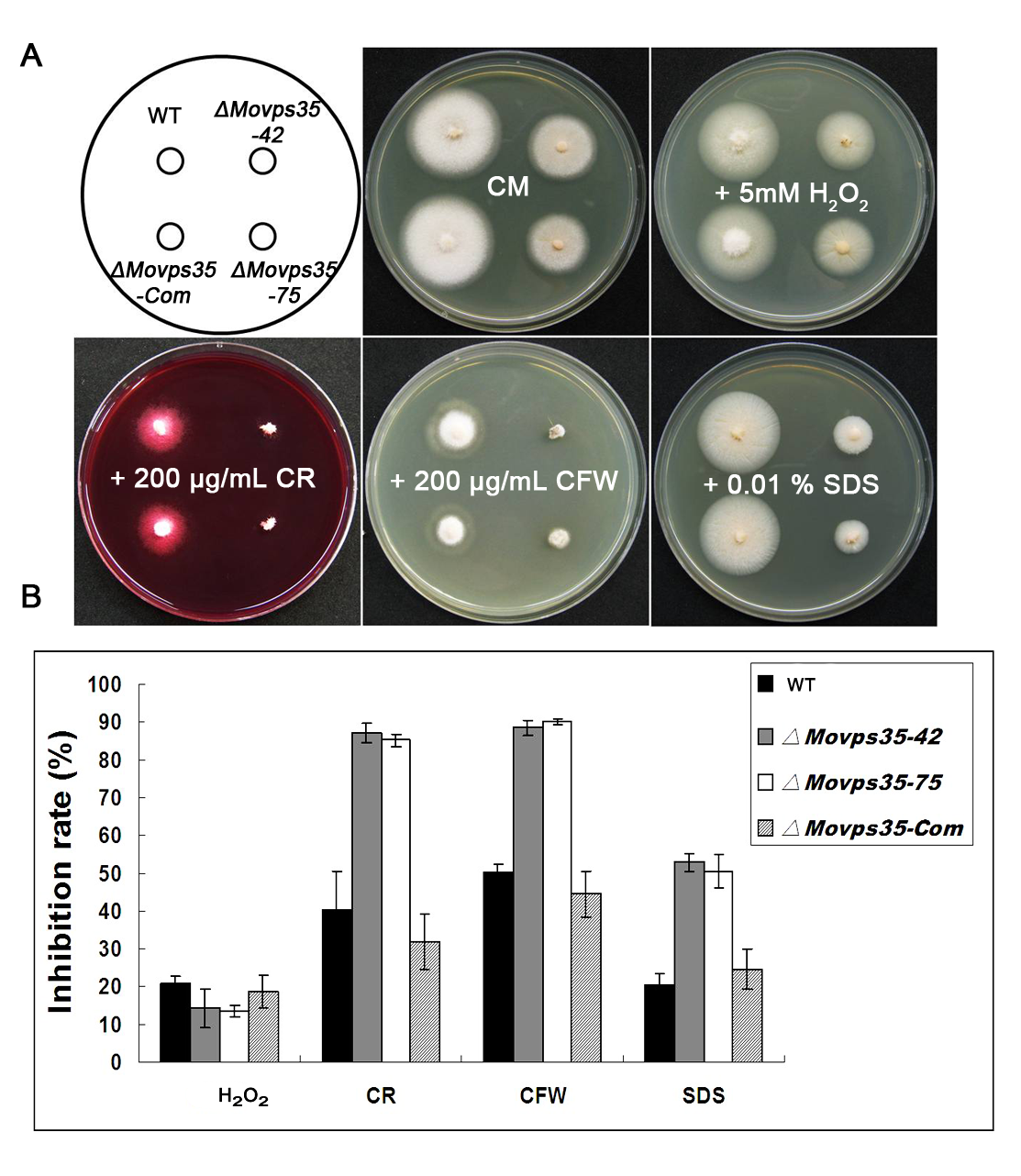

Supplement: S2 Fig — (A) The wild type, ΔMovps35 and the complemented strain were incubated on CM medium supplemented with various stress inducers for 6 days at 28 0C. (B) Analysis of the growth inhibition rate in mycelia in CM supplemented with various stress inducers. Data comprise three independent experiments with triple replications in each instance. (TIF) [file pgen.1005704.s002.tif]

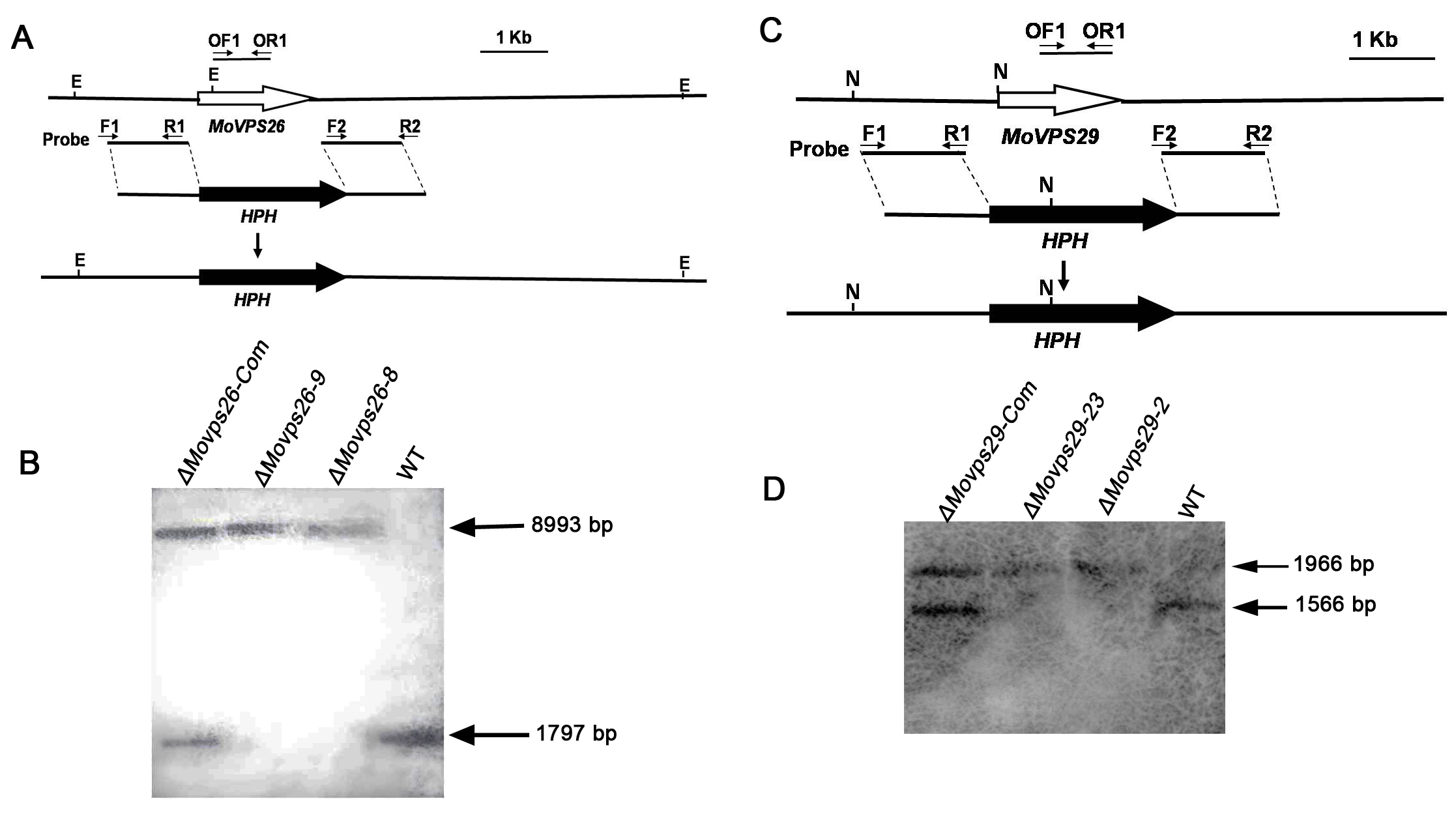

Supplement: S3 Fig — (A) Schematic diagram of the genomic region of the MoVPS26 and HPH genes. Primers F1 (MO04830AF), R1 (MO04830AR), F2 (MO04830BF) and R2 (MO04830BR) were used to generate MoVPS35 gene replacement constructs, and OF1 (MO04830OF), OR1 (MO04830OR), F1 (MO04830AF) and R1 (MO04830AR) were used for mutant screening and identification. E, EcoRV. (B) DNA gel blots of EcoRV -digested genomic DNA were hybridized with MoVPS26 upstream fragment as the probe. A 1.797-kb band was observed in the wild type and complementation strain, while 8.993-kb bands were observed in the two independent mutants and complementation strain. ΔKu70, wild-type strain, ΔMovps26-9 and ΔMovps26-8, null mutant, ΔMovps26-Com, complementation strain. (C) Schematic diagram of the genomic region of the MoVPS29 and HPH genes. Primers F1 (MO02524AF), R1 (MO02524AR), F2 (MO02524BF) and R2 (MO02524BR) were used to generate MoVPS29 gene replacement constructs, and OF1 (MO02524OF), OR1 (MO02524OR), F1 (MO02524AF) and R1 (MO02524AR) were used for mutant screening and identification. N, Nco I. (D) DNA gel blots of Nco I-digested genomic DNA were hybridized with MoVPS29 upstream fragment as the probe. A 1.566-kb band was observed in the wild type and complementation strain, while 1.966-kb bands were observed in the two independent mutants and complementation strain. ΔKu70, wild-type strain, ΔMovps29-2 and ΔMovps29-23, null mutant, ΔMovps29-Com, complementation strain. (TIF) [file pgen.1005704.s003.tif]

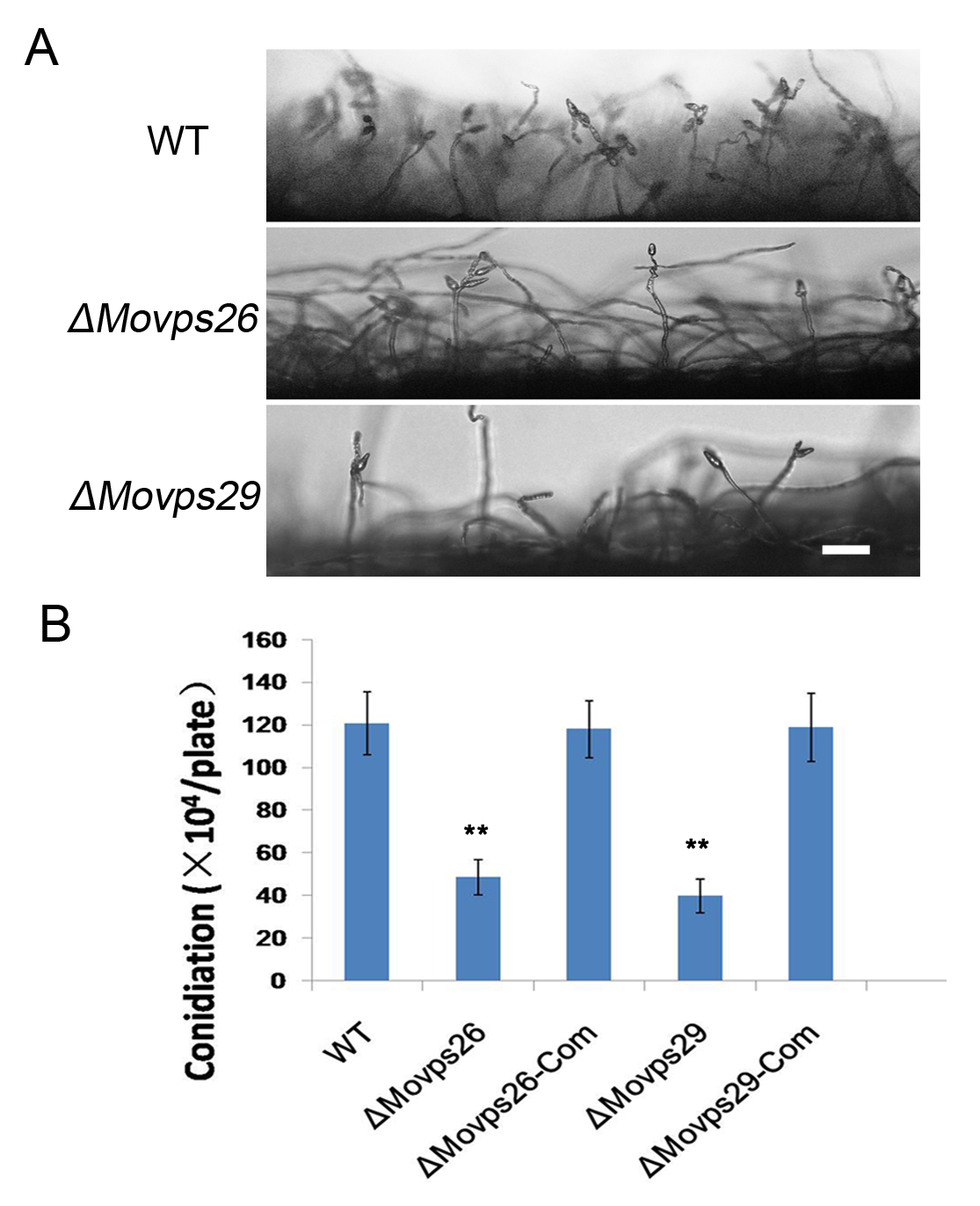

Supplement: S4 Fig — (A) Development of conidia on conidiophores was significantly reduced in ΔMovps26 and ΔMovps29 mutants. Scale bar = 50 μm. (B) Analysis of conidia production. The respective strains were initially grown in the dark for a day followed by exposure to constant illumination for 14 day on RPA plate (diameter 9 cm). Data represents mean ± SD based on three independent replicates, and double asterisks indicate statistically significant differences (P < 0.01). (TIF) [file pgen.1005704.s004.tif]

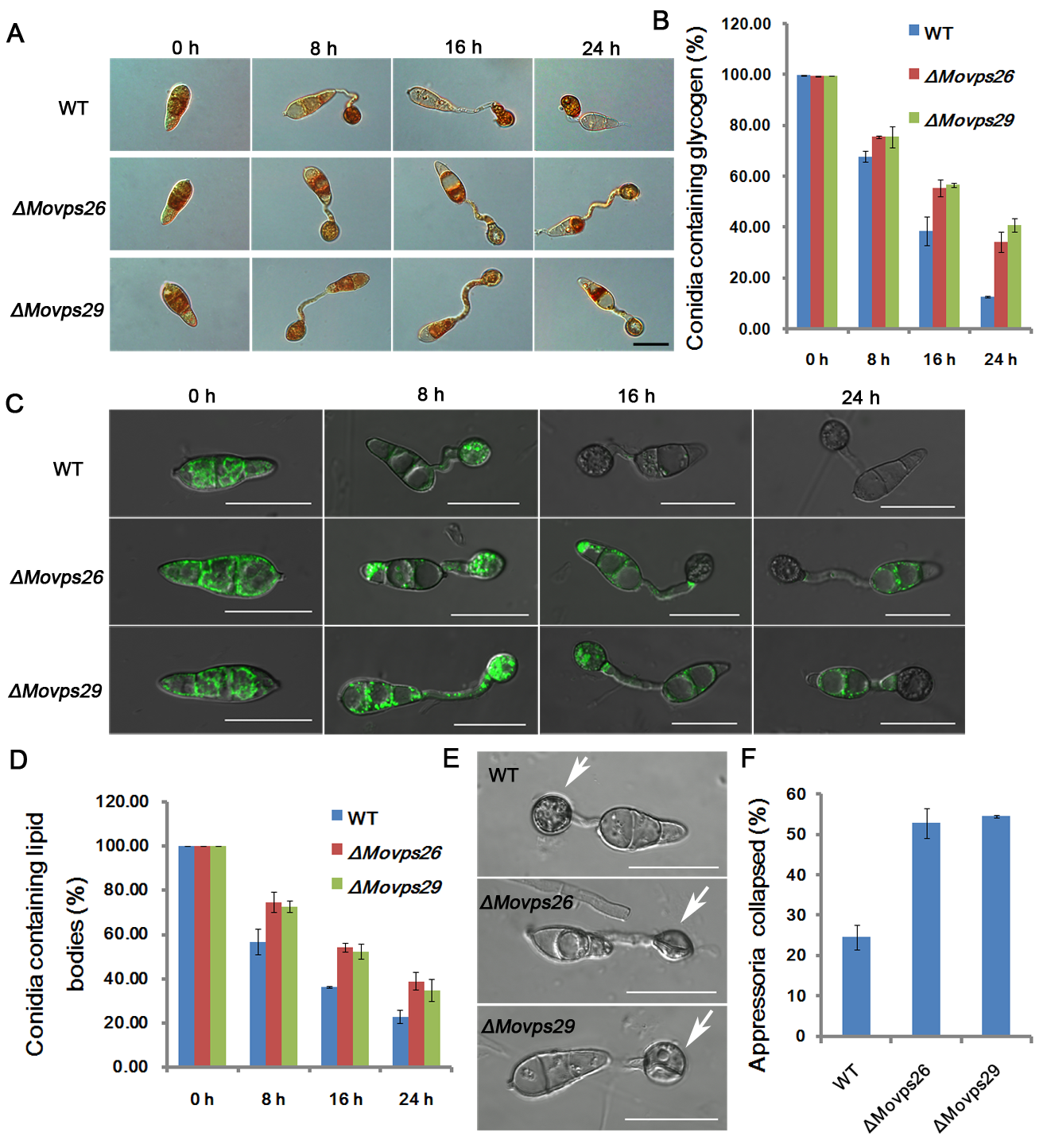

Supplement: S5 Fig — (A) Conidia from wild type (WT), ΔMovps26 or ΔMovps26 strain were germinated on hydrophobic gelbond membranes. Drop of water was replaced by iodine solution at 0 h, 8 h, 16 h and 24 h to stain for and microscopically visualize glycogen as yellowish-brown deposits. (B) Quantitation of the total glycogen content in conidia during pathogenic development in the indicated strains. (C) MoVps26 and MoVps29 are involved in lipid body translocation and degradation during appressorium morphogenesis. Conidia of M. oryzae WT, ΔMovps26 and ΔMovps29 were incubated in water droplets on the hydrophobic surface of gelbond and allowed to form appressoria for up to 24 h. Samples were removed at 0, 8, 16 and 24 h and stained with Bodipy to visualize lipid bodies by confocal microscopy. (D) Quantitative analysis of lipid bodies during appressorium morphogenesis. The bar charts show the mean and standard deviation from three independent replicates of the experiment. (E) Appressorium turgor was measured by incipient cytorrhysis assays. Appressoria were allowed to form on plastic coverslips for 24 h, and the collapsed appressoria assessed after exposure to 2 M glycerol solutions. White arrows indicate the appressoria in the WT, ΔMovps26 or ΔMovps26 strain. (F) Proportion of collapsed appressoria after exposure of conidia to 2 M glycerol. Bars = 20 μm. (TIF) [file pgen.1005704.s005.tif]

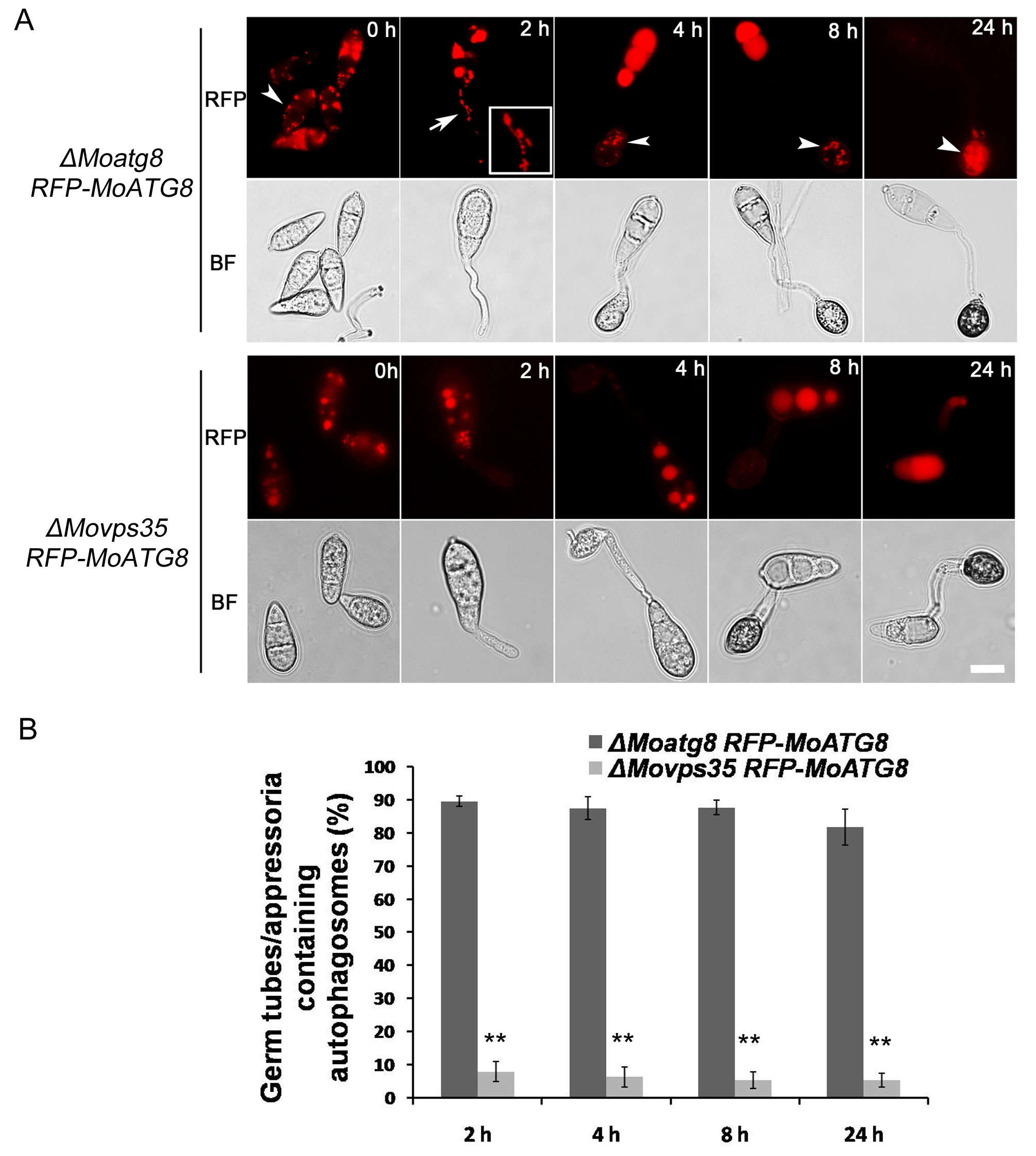

Supplement: S6 Fig — (A) Conidia from RFP-MoAtg8 expressed in the ΔMoatg8 or ΔMovps35 strain were inoculated onto glass coverslips and observed by epifluorescence microscopy at the indicated times. RFP-MoAtg8 punctate structures (arrows and arrowheads) were significantly reduced in number in both germ tubes and appressoria of ΔMovps35 RFP-MoATG8 when compared to ΔMoatg8 RFP-MoATG8. Bar = 10 μm. (B) Bar chart showing ratio of punctate autophagosomes present in germ tubes and appressoria 2 h, 4 h, 8 h and 24 h after inoculation. Values represent mean and standard deviation from three independent replicates using 200 conidia per sample. Double asterisks indicate statistically significant differences (P < 0.01). (TIF) [file pgen.1005704.s006.tif]

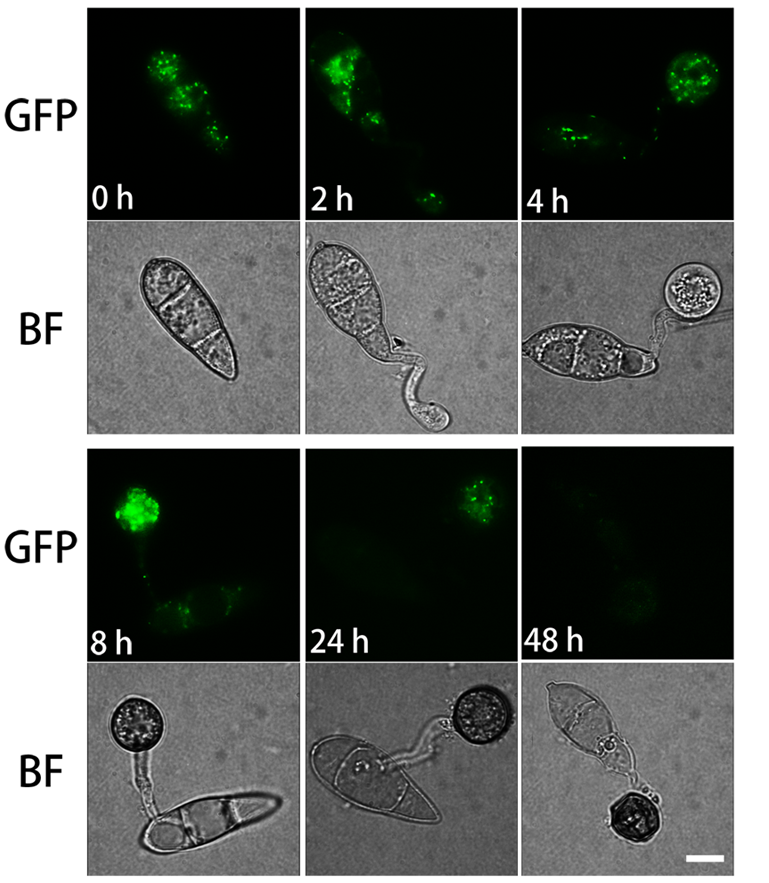

Supplement: S7 Fig — Conidia were allowed to form appressoria and localization of MoVps35-GFP observed during 48 h using an Olympus BX-51 epifluorescence microscope. Fusion proteins localized to cytoplasm as small punctae in the conidium, germ tube and appressorium. BF = Bright field. Scale bar = 10 μm. (TIF) [file pgen.1005704.s007.tif]

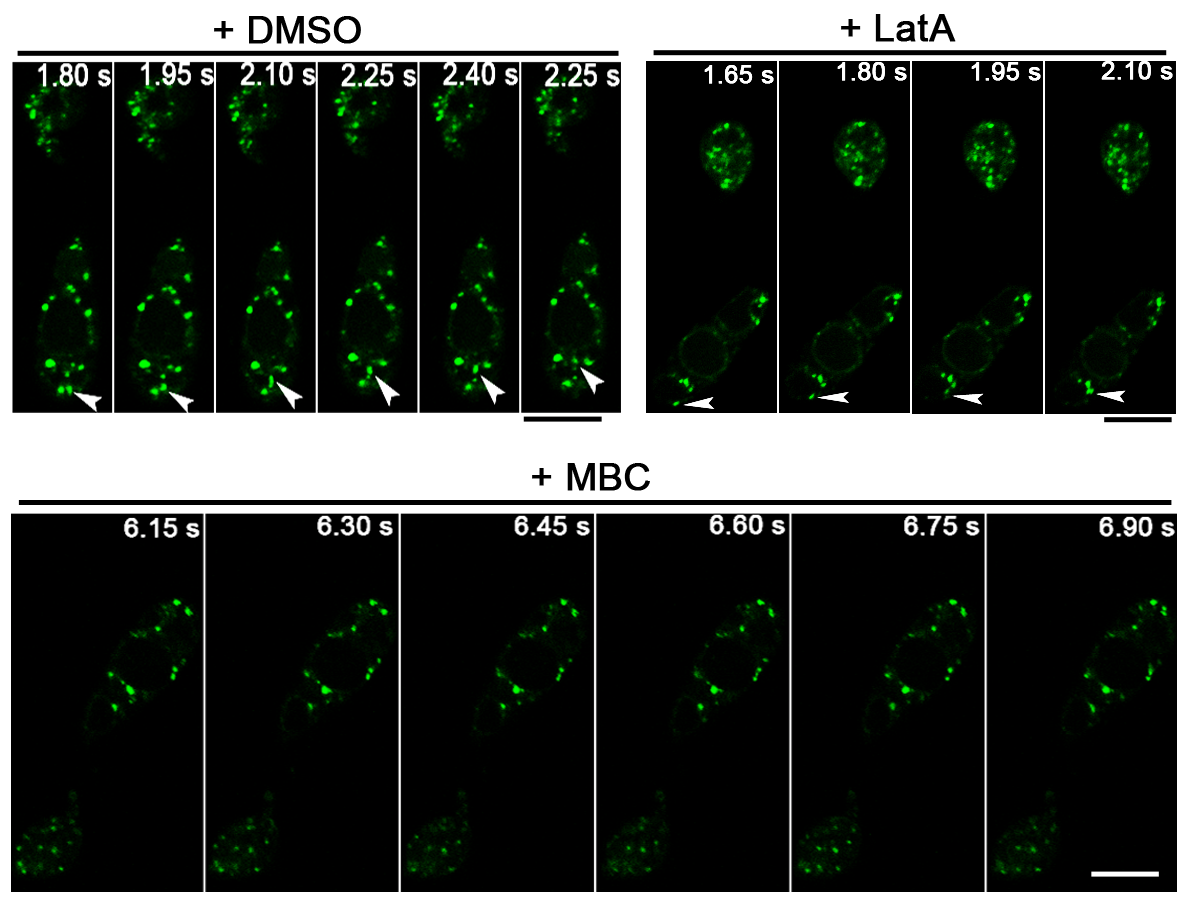

Supplement: S8 Fig — Actin polymerization inhibitor LatA and microtubule cytoskeleton inhibitor MBC were added to developing appressoria, respectively. Fluorescently labeled motile punctate compartments lost the ability to carry out long distance transport in fungal cells treated with MBC. The mobility of MoVps35-GFP was not visibly impaired after treatment with LatA. DMSO-treated sample served as a control. Arrowheads indicate the relative position of punctate compartments at each time point. Elapsed time indicated in seconds. See also S4–S6 Movies. Scale bars = 10 μm. (TIF) [file pgen.1005704.s008.tif]

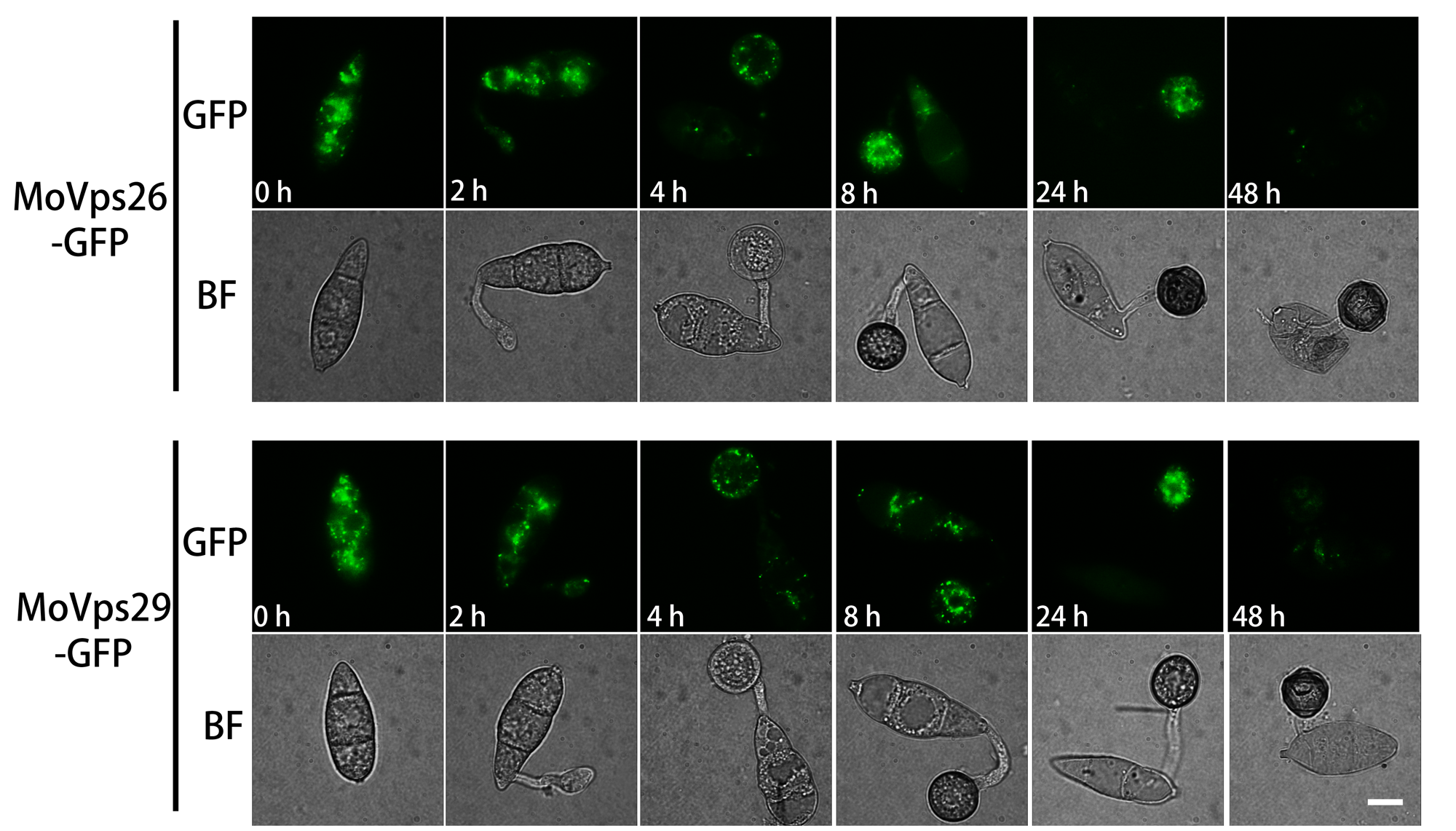

Supplement: S9 Fig — Conidia were allowed to form appressoria and localization of MoVps26-GFP or MoVps29-GFP observed during 48 h using an Olympus BX-51 epifluorescence microscope. Fusion proteins were apparent as small cytosolic punctae in conidia, germ tubes and appressoria. BF = Bright field. Scale bar = 10 μm. (TIF) [file pgen.1005704.s009.tif]

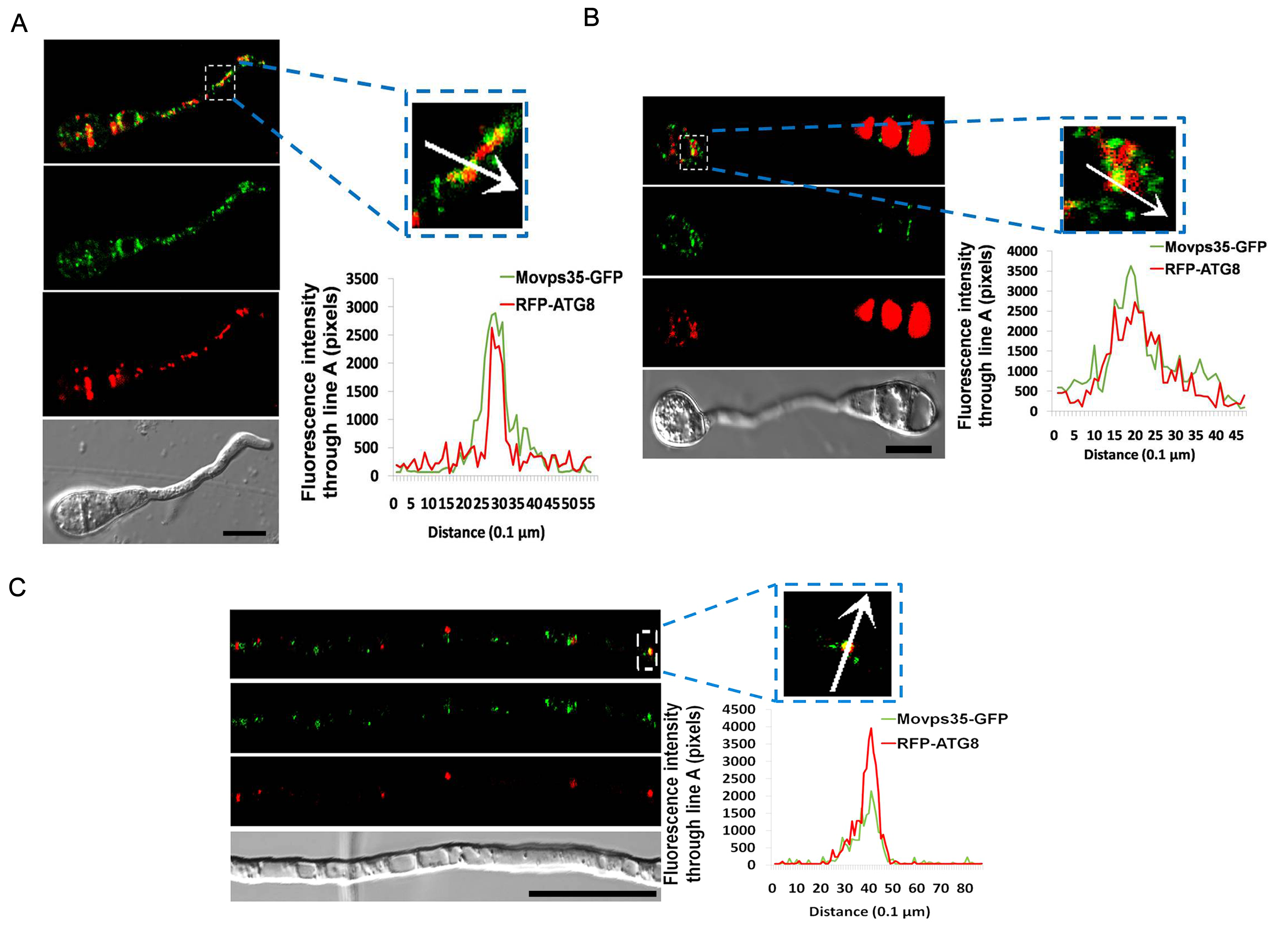

Supplement: S10 Fig — Live cell imaging of distribution and dynamics of MoVps35-GFP and RFP-MoAtg8 pathogenic and vegetative growth in M. oryzae. The dotted box (left panel) and enlarged dotted box (right panel) highlight compartments showing co-localized MoVps35-GFP and RFP-MoAtg8. White arrow in the inset shows the path for fluorescence intensity distribution by line-scan analysis. Images on the left panel are merged MoVps35-GFP (green) and RFP-MoAtg8 (red), MoVps35-GFP (green) alone, RFP-MoAtg8 (red) alone, and DIC. Bar = 10 μm. (A) conidial germination stage. (B) appressorium initiation. (C) mycelial growth under nitrogen stravation condition. (TIF) [file pgen.1005704.s010.tif]

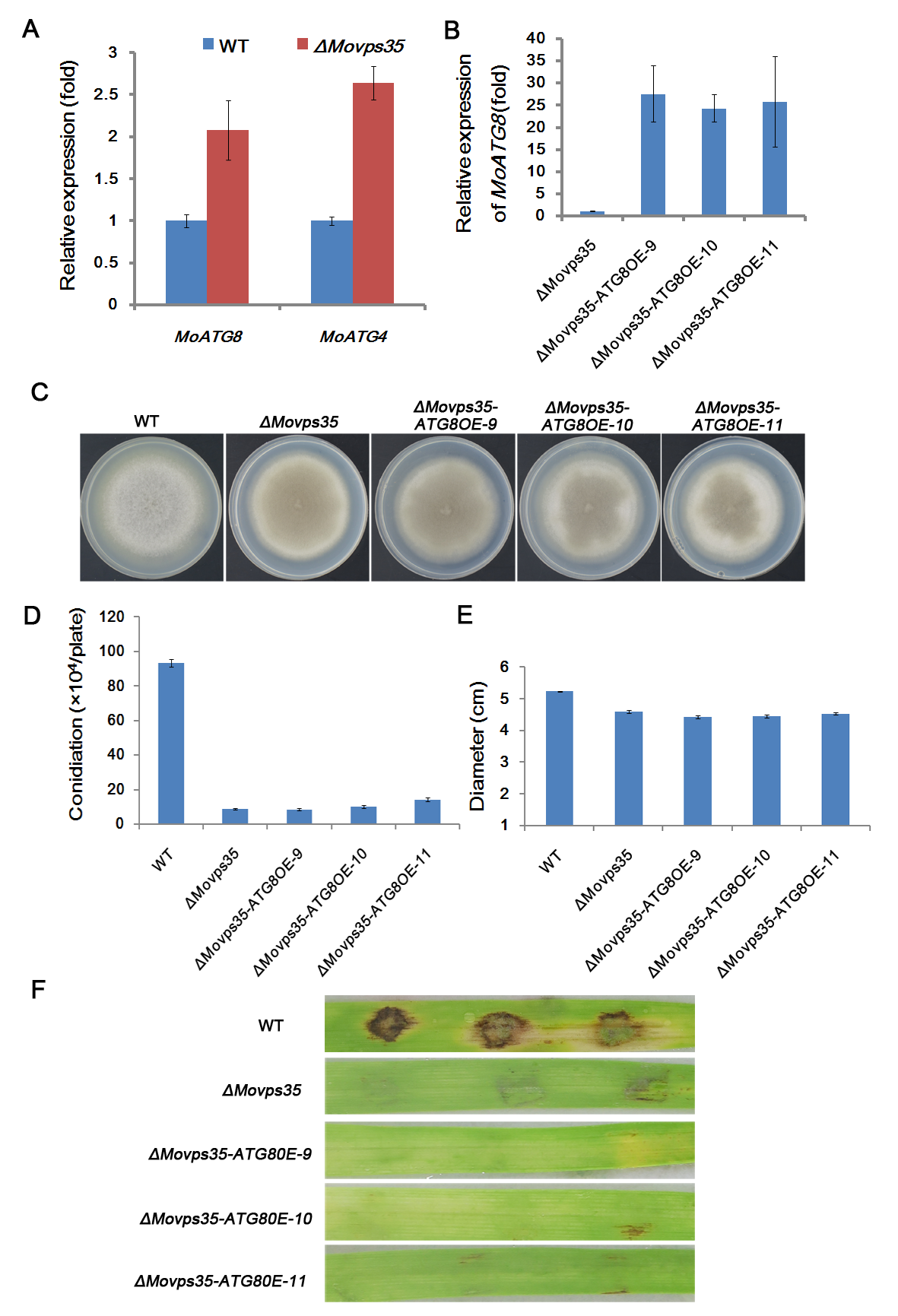

Supplement: S11 Fig — (A) The expression levels of MoATG8 and MoATG4 genes were assayed by qRT-PCR with RNA isolated from vegetative hyphae culturing on MM-N medium. (B) qRT-PCR-based confirmation of MoATG8 transcript levels in ΔMovps35-ATG8OE strains. (C) Morphology of the colonies of wild type, ΔMovps35 and ΔMovps35-ATG8OE strain. Analysis of conidiation (D), diameter of the colonies (E) and pathogenicity (F) in wild type, ΔMovps35 and ΔMovps35-ATG8OE strain. (TIF) [file pgen.1005704.s011.tif]
