# Supplementary material for: Retromer Is Essential for Autophagy-Dependent Plant Infection by the Rice Blast Fungus
Source: PLoS Genet. 2015 Dec 10;11(12):e1005704. doi: 10.1371/journal.pgen.1005704 (PMC4686016; doi:10.1371/journal.pgen.1005704)
Supplement: S1 Table — (DOCX) [file pgen.1005704.s012.docx]

**Supplemental Table 1.** Putative retromer components were identified by Blastp analysis

| *Saccharomyces cerevisiae* | *Magnaporthe*  *oryzae* | Query coverage | E value | Max identity |
| --- | --- | --- | --- | --- |
| *VPS35* | MGG_05089  *(MoVPS35)* | 80% | 1e-94 | 57% |
| *VPS26* | MGG_04830  *(MoVPS26)* | 88% | 1e-53 | 64% |
| *VPS29* | MGG_02524  *(MoVPS29)* | 89% | 4e-35 | 49% |
| *VPS17* | MGG_01434  *(MoVPS17)* | 81% | 4e-92 | 35% |
| *VPS5* | MGG_06743  *(MoVPS5)* | 59% | 7e-65 | 33% |
