# Supplementary material for: Retromer Is Essential for Autophagy-Dependent Plant Infection by the Rice Blast Fungus
Source: PLoS Genet. 2015 Dec 10;11(12):e1005704. doi: 10.1371/journal.pgen.1005704 (PMC4686016; doi:10.1371/journal.pgen.1005704)
Supplement: S2 Table — (DOC) [file pgen.1005704.s013.doc]

**Supplemental Table 2**. Wild-type and mutant strains of fungi used in this study

| **Strain** | **Genotype description** | **Reference** |
| --- | --- | --- |
| *Δku70* | *ΔMoku70* deletion mutant of Guy11 |  |
| *ΔMovps35* | MGG_05089 deletion mutant in *ΔMoku70* background | This study |
| *ΔMovps35-Com* | *ΔMovps35* strain expressing the MoVps35-GFP construct | This study |
| WT:His-GFP | *Δku70* strain expressing Histone H1 encoding gene (H1) from *Neurospora crassa*, fused to GFP | This study |
| *ΔMovps35:His-GFP* | *ΔMovps35* strain expressing Histone H1 encoding gene (H1) from *Neurospora crassa*, fused to GFP | This study |
| *ΔMoatg8:RFP-ATG8* | *ΔMoatg8* strain expressing the RFP-ATG8 construct |  |
| *ΔMovps35:RFP-ATG8* | *ΔMovps35* strain expressing the RFP-ATG8 construct | This study |
| *ΔMoatg8:RFP-ATG8 MoVps35-GFP* | *ΔMoatg8:RFP-ATG8* strain expressing the MoVps35-GFP construct | This study |
| *ΔMovps26* | MGG_04830 deletion mutant in *ΔMoku70* background | This study |
| *ΔMovps26-Com* | *ΔMovps26* strain expressing the MoVps26-GFP construct | This study |
| *ΔMovps29* | MGG_02524 deletion mutant in *ΔMoku70* background | This study |
| *ΔMovps29-Com* | *ΔMovps29* strain expressing the MoVps29-GFP construct | This study |

**Deng, Y.Z., Ramos-Pamplona, M., and Naqvi, N.I.** (2009). Autophagy-assisted glycogen catabolism regulates asexual differentiation in Magnaporthe oryzae. Autophagy **5,** 33-43.

**Kershaw, M.J., and Talbot, N.J.** (2009). Genome-wide functional analysis reveals that infection-associated fungal autophagy is necessary for rice blast disease. Proceedings of the National Academy of Sciences of the United States of America **106,** 15967-15972.
