# Supplementary material for: Retromer Is Essential for Autophagy-Dependent Plant Infection by the Rice Blast Fungus
Source: PLoS Genet. 2015 Dec 10;11(12):e1005704. doi: 10.1371/journal.pgen.1005704 (PMC4686016; doi:10.1371/journal.pgen.1005704)
Supplement: S3 Table — (DOC) [file pgen.1005704.s014.doc]

**Supplemental Table 3.** PCR primers used in this study.

| **Primers** | **Sequence(5’-3’)** | **Application** |
| --- | --- | --- |
| MO05089AF | CCGCTCGAGGGCTTCAAAGTGTTATAGGG | *MoVPS35* deletion and probe |
| MO05089AR | CCGGAATTCTGGTTGGTGGTTCAAGGTA |
| MO05089BF | CGGGATCCAGCGTTGGAGTTGGTGAA |
| MO05089BR | GCTCTAGAAAGGCAGAGGATGGAGTTTA |
| MO05089OF | AGCAACAGGAGCCAGTAAA | *∆Movps35* mutant screen |
| MO05089OR | GATTGCGAGGACCAGTTAT |
| MO05089UA | AGATGCAGACCGACTCCT | *∆Movps35* mutant screen |
| H853 | GACAGACGTCGCGGTGAGTT |
| MO05089CF | TGGCAGATGCTTCTCACTCAG | *∆Movps35* complementation and GFP fusion |
| MO05089CR | GAAGATCTCTTGGGATCCAACACAATTCC |
| Bgl II-GFPF | GAAGATCTATGGTGAGCAAGGGCGAGGAGC | GFP fusion |
| Bgl II-GFPR | GAAGATCTGTGGAGATGTGGAGTGGGCGCTT |
| RFP-For | CATATGGACAACACCGAGGACGT | RFP-ATG8 screen |
| Atg8-Rev | GGTATCCACTCCGCGTCGACAGACGA |
| MO05089BDF | CATGCCATGGAGATGGCGTCGGTCCCAGCTC | Y2H BD vector |
| MO05089BDR | CATGCCATGGTCACTTGGGATCCAACACAATTCC |
| MO04830ADF | CCGGAATTCATGTCCTACTTCTTCTCGACCCCAG | Y2H AD vector |
| MO04830ADR | CGCGGATCCCTACGCAGGGACCGCCTGTATC |
| MO02524ADF | CCGGAATTCATGGCCTTCCTAATCCTGGTC | Y2H AD vector |
| MO02524ADR | CGCGGATCCTCATGATGCCGAAGCTGTTG |
| MO04830AF | GTGGCTGTGGTGGTTGTGA | *MoVPS26* deletion and probe |
| MO04830AR | TTGACCTCCACTAGCTCCAGCCAAGCCAAGTAGCTTGTATCCCCGCG |
| MO04830BF | GAATAGAGTAGATGCCGACCGCGGGTTCGGCTCTATGCTTCCTCTT |
| MO04830BR | CGTCGGTGATGATGTTCTC |
| HYG/F | GGCTTGGCTGGAGCTAGTGGAGGTCAA | Gene deletion |
| HY/R | GTATTGACCGATTCCTTGCGGTCCGAA |
| YG/F | GATGTAGGAGGGCGTGGATATGTCCT |
| HYG/R | AACCCGCGGTCGGCATCTACTCTATTC |
| MO04830OF | GGAATCAAGGTGCAGTTCATCG | *∆Movps26* screen |
| MO04830OR | GGGACCGCCTGTATCCTACTCT |
| MO04830UA | GTCGTTCGGGTCGTAAATCG | *∆Movps26* screen |
| MO04830CF | CCGTTGATGAACCCATTGCCACTC | *∆Movps26* complementation and GFP fusion |
| MO04830CR | GGACTAGTCGCAGGGACCGCCTGTATCCTACTC |
| speI-GFPF | GGACTAGTAGCAAGGGCGAGGAGCTGT | GFP fusion |
| speI-GFPR | GGACTAGTCTGTGCATTCTGGGTAAACGA |
| MO02524AF | CTTCTCAATCTCGGGCAGTT | *MoVPS29* deletion and probe |
| MO02524AR | TTGACCTCCACTAGCTCCAGCCAAGCCGTGGAGCTTACACGACAGG |
| MO02524BF | GAATAGAGTAGATGCCGACCGCGGGTTACAATGCTTTTGCTCGTGG |
| MO02524BR | GCAAAGAGCACGAATGAGGA |
| MO02524OF | GCTCCAACAGGCTACCTAACA | *∆Movps29* screen |
| MO02524OR | GCGGCTCGACTACTTTCGT |
| MO02524UA | GCAATCTCCTCAGAAGCCAC | *∆Movps29* screen |
| MO02524CF | GGGGTAGCAAAGTATGAAGGC | *∆Movps29* complementation and GFP fusion |
| MO02524CR | GGACTAGTTGATGCCGAAGCTGTTGTT |
| Tubulin QF | TCTGACTTCAGGAATGGTCGTTAC | qRT-PCR |
| Tubulin QR | AGCGGTCTGGATGTTGTTGG |
| MoATG4QF | GTTCAGGGCAAGTCCATCATTA | qRT-PCR |
| MoATG4QR | CGGTATCGCCATCATCGTC |
| MoATG8QF | TCGGATTGCCACGGTCAC | qRT-PCR |
| MoATG8QR | GCACGGTTCCAAACTTCAGG |
